# Secure quantum key distribution against correlated leakage source

**DOI:** 10.1126/sciadv.aed2420

**Published:** 2026-04-10

**Authors:** Jia-Xuan Li, Yang-Guang Shan, Rong Wang, Feng-Yu Lu, Zhen-Qiang Yin, Shuang Wang, Wei Chen, De-Yong He, Guang-Can Guo, Zheng-Fu Han

**Affiliations:** ^1^ Laboratory of Quantum Information, University of Science and Technology of China, Hefei, Anhui 230026, P. R. China.; ^2^ CAS Center for Excellence in Quantum Information and Quantum Physics, University of Science and Technology of China, Hefei, Anhui 230026, P. R. China.; ^3^ Anhui Province Key Laboratory of Quantum Network, University of Science and Technology of China, Hefei, Anhui 230026, P. R. China.; ^4^ School of Cyberspace, Hangzhou Dianzi University, Hangzhou, Zhejiang 310018, P. R. China.; ^5^ Hefei National Laboratory, University of Science and Technology of China, Hefei 230088, P. R. China.

## Abstract

Quantum key distribution (QKD) provides information-theoretic security based on quantum mechanics, yet its implementation is hindered by source imperfections. Among these, correlations between transmitted pulses present a critical but underexplored threat to QKD’s theoretical security. We propose a general framework for QKD security under correlated sources, achieving the first finite-key analysis by extending and reorganizing QKD rounds using the generalized chain rule. Inspired by side-channel-secure QKD, we design a protocol secure against correlated leakage sources, requiring only the correlation range and lower bounds on the vacuum component of the prepared states. Our framework also applies to other QKD protocols, providing a general approach to address correlation-induced vulnerabilities. Simulations demonstrate the effectiveness of our protocol and its significantly superior tolerance to imperfections compared to existing protocols. This work provides a crucial step toward closing security loopholes in QKD, enhancing its practicality, and ensuring long-distance, high-performance secure communication under real-world constraints.

## Teaser

Presenting a general finite-key framework and a secure QKD protocol, closing source correlation loopholes.

## INTRODUCTION

With the rapid development and widespread adoption of the internet, information security has become one of the most critical issues in modern society. The modern cryptography, as the most widely used approach, ensures data privacy and security, but its security relies on computational complexity (*1*), which is threatened by advancing computing power, especially quantum computing (*2*, *3*). As countermeasures to the threats faced by modern cryptography, postquantum cryptography and quantum key distribution (QKD) (*4*) have been proposed. Among them, QKD is not based on computational hardness but instead relies on the fundamental principles of physics and, as a form of symmetric cryptography, can achieve theoretical security under appropriate assumptions. Consequently, QKD has attracted substantial attention as one of the major directions in the development of modern cryptography. QKD uses the principles of quantum mechanics to achieve information theoretic security in key agreement (*5*–*8*), which means that theoretically, even an eavesdropper with unlimited computational power cannot break its security. However, the theoretical security of QKD still faces challenges in practical deployments, especially when considering imperfections in real-world devices. Fortunately, all vulnerabilities related to measurement devices can be addressed using measurement device–independent QKD (MDI QKD) (*9*–*11*), as well as variants such as twin-field QKD (TF QKD) (*12*–*15*) and mode-pairing QKD (MP QKD) (*16*). However, the imperfections of the source still introduce potential security loopholes in practical QKD implementations, which require further resolution.

The security loopholes caused by imperfect sources primarily stem from three aspects (*17*): state preparation flaws (SPFs) due to the limited modulation accuracy of the device, information leakage caused by side channels, and information leakage resulting from classical correlations between pulses. Here, we refer to a source exhibiting these three types of imperfections as correlated leakage source. Fortunately, several effective solutions for the SPF problem already exist (*18*, *19*). Moreover, based on the resolution of SPF, multiple approaches have also been proposed to address the side-channel issue (*20*–*27*). In particular, a recently proposed protocol, known as side-channel-secure QKD (SCS QKD) (*28*–*31*), is based on the sending-or-not-sending QKD (SNS QKD) (*14*) scheme. By imposing a lower bound constraint on the vacuum component of the transmitted states, SCS QKD is immune to all information leakage caused by any unknown side channels. Compared to device-independent QKD (*32*), SCS QKD is considered the only protocol secured under side channel that can be implemented with commercial devices while achieving long-distance transmission (*33*).

However, compared to the well-studied side-channel problem, the impact of correlations on security remains less thoroughly investigated, leaving the QKD systems not completely secure under correlated leakage source. Now, the primary approaches for handling correlations include the postselection methods (*34*–*36*) and enhancing protocols (*17*, *37*–*42*), using mathematical techniques such as reference techniques (*17*) and quantum coin (*42*). Nevertheless, these methods have notable limitations, such as the inability of some protocols to handle high-order correlations, the need for complex modeling of the magnitude of correlation, and poor tolerance to existing device parameters. These factors make handling correlations in practice a challenging task, and only a few experiments have addressed the correlation problem for certain parameters (*41*). Moreover, none of these existing protocols provide a complete finite-key analysis, limiting their applicability in real-world QKD systems.

To address these challenges, this paper makes two main contributions. First, we propose a security analysis framework for QKD under correlation. By extending and rearranging QKD rounds, using the generalized chain-rule result (*43*), we establish security constraints that allow for finite-key analysis in the presence of correlations.

This security analysis framework applies to a broad class of protocols ranging from BB84 (*4*–*8*) to TF QKD (*12*–*15*), allowing correlation issues to be solved as the well-studied side-channel problems and providing a systematic approach toward fully resolving such issues. Second, we introduce a secure QKD against correlated leakage source based on the two-state SNS QKD (*28*–*31*). This protocol only assumes a bounded correlation range and a lower bound on the vacuum component of the prepared states, enabling secure key generation even in the presence of SPFs, side channels, and correlations. Compared to existing protocols, our security analysis framework is the first to enable finite-length analysis under correlation conditions, and this framework is not only applicable to our protocol but can also be easily extended to any protocol involving correlations. Moreover, our protocol does not require any characterization of the magnitude or the specific form of side channels and correlations. It can tolerate practical device imperfections and demonstrates strong robustness against high-order correlations. Simulation results show that our protocol can efficiently generate keys under realistic device parameters. When the correlation range is 5, it only loses 10 dB of the maximum attenuation, and it can tolerate a correlation range as high as 1000, far exceeding the maximum correlation range of 6 observed in existing experiments (*44*). This substantially enhances the security and practical feasibility of QKD systems under realistic physical constraints, marking a crucial step toward achieving loophole-free and high-performance QKD.

## RESULTS

### Security analysis framework addressing correlated sources

In experiments, as summarized in Table 1, correlations are commonly observed and can be characterized in terms of their range, form, and magnitude. Due to correlation, one of the most critical assumptions in QKD security proofs, independent distributed state preparation, is violated. This substantially complicates the security analysis, especially under finite-key conditions. In this work, we use the generalized chain-rule result (*43*) to prove the security equivalence between correlated sources and uncorrelated sources, effectively establishing a reduction from nonindependent scenarios to the independent case. Furthermore, through additional discussion, this equivalence can be extended to prove that any protocol with correlated leakage source, including correlations, side channels, and SPFs, can find a security bound to an independent and identically distributed (i.i.d.) protocol.

**Table 1.** Current experimental measurement results on correlation of the real QKD systems. Range, correlation range, the maximum range to which the setting of one round can influence other rounds; Form, the specific form and dimensionality in which correlations manifest; Magnitude, the strength of the correlations, typically defined in terms of the relative deviation induced by correlations in quantities such as the inner product of quantum states or intensity.

Range	1 (*36*, *45*, *46*)	2 (*47*)	3 (*41*, *48*–*50*)	6 (*44*)
Form	Intensity	Intensity	Intensity (*41*, *48*–*50*), phase (*49*), state (*50*)	Intensity
Magnitude	$O\left(10^{-1}\right)$ (*36*), $O\left(10^{-2}\right)$ (*45*, *46*)	$O\left(10^{-2}\right)$	$O\left(10^{-2}\right)$ (*41*, *48*, *49*), $O\left(10^{-3}\right)$ (*50*)	$O\left(10^{-2}\right)$

First, we give our basic assumption. We assume that the range of correlation is limited (*17*, *36*–*41*). The correlation range refers to the maximum number of previous rounds whose settings can influence the state preparation in a given round. This assumption has been validated as reasonable by some experiments (*36*, *41*, *44*–*50*). Thus, we introduce the first assumption.

Assumption 1. *The correlation is constrained within a maximum range* ξ*.*

The security of a QKD protocol can be characterized by the conditional smooth min-entropy $H_{\text{min}}^{\epsilon }{\left(Z_{A}|E^{\prime }\right)}_{\rho }$, where $Z_{A}$ refers to the raw key of Alice, $E^{\prime }$ refers to the uncertainty system of the eavesdropper Eve, and ρ represents the quantum state shared in the protocol, which includes Eve’s optimal attack system.

We divide $Z_{A}$ into several subsets $Z_{A}=Z_{A_{1}}Z_{A_{2}}\ldots Z_{A_{\xi +1}}$, where $Z_{A_{i}}$ represents the key sequence generated in the kth rounds of the protocol, where $k\in \left\{i+n\left(\xi +1\right)|n\in {\mathbb{N}}_{0},i+n\left(\xi +1\right)\leq N\right\}$.

Because the correlation range ξ ensures that two keys separated by ξ rounds do not influence each other, $Z_{A_{i}}$ and $Z_{A_{j}}$ are independent from each other if i≠j. By the generalized chain-rule result (*43*) and data processing inequality, we can use $H_{\text{min}}^{\epsilon_{i}}{\left(Z_{A_{i}}|\left({∁}_{Z_{A}}Z_{A_{i}}\right)E^{\prime }\right)}_{\rho }$ to estimate a lower bound for $H_{\text{min}}^{\epsilon }{\left(Z_{A}|E^{\prime }\right)}_{\rho }$, where $\left({∁}_{Z_{A}}Z_{A_{i}}\right)$ represents the part of set $Z_{A}$ excluding $Z_{A_{i}}$, denoted as $Z_{A_{1}}Z_{A_{2}}\ldots Z_{A_{i-1}}Z_{A_{i+1}}\ldots Z_{A_{\xi +1}}$.

**Fig. 1.**
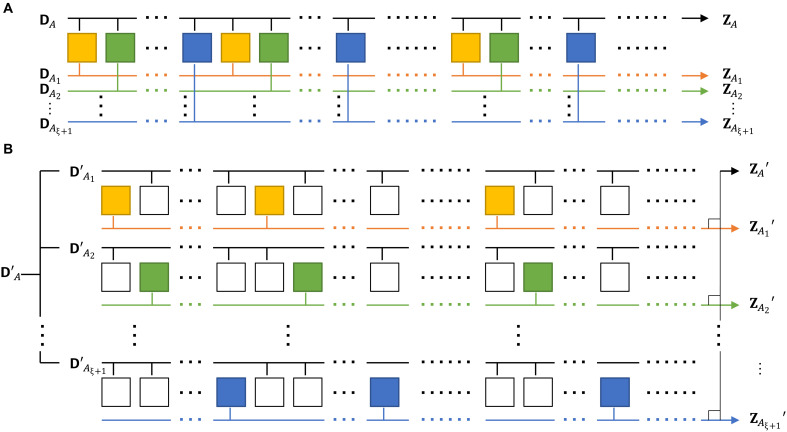
Schematic diagrams of the original and new protocols. (**A**) Each square represents a single round, with yellow, green, …, and blue indicating rounds mod(ξ+1)=1,2,…,(ξ+1) (or 0); the rightward arrow represents the process of filtering raw key bits. The protocol proceeds from left to right for a total of N rounds, with each category being executed $N_{i}$ rounds. The definitions of D and Z are detailed in the “Security analysis framework addressing correlated sources” section. (**B**) Each square represents a single round, where colored squares indicate “key generation rounds,” and uncolored squares represent rounds where data are disclosed. Each pair of adjacent colored rounds is separated by ξ data disclosure rounds. Yellow, green, …, and blue correspond one to one with the rounds of the same colors in (A). The protocol proceeds from left to right, moving to the next row after completing one. A total of $N^{\prime }=N$ key generation rounds are executed, which corresponds to $\left(\xi +1\right)N^{\prime }$ rounds in total, with each category being executed $N_{i}^{\prime }=N_{i}$ rounds. The definitions of $D^{\prime }$ and $Z^{\prime }$ are detailed in the “Security analysis framework addressing correlated sources” section.

Furthermore, we can relax the condition ${∁}_{Z_{A}}Z_{A_{i}}$ in $H_{\text{min}}^{\epsilon_{i}}{\left(Z_{A_{i}}|\left({∁}_{Z_{A}}Z_{A_{i}}\right)E^{\prime }\right)}_{\rho }$ even more. Define all of Alice’s local systems [including the raw key, (potential) basis selection, intensity selection, as well as ancillas control random drifts and fluctuations] as $D_{A}$, and, similar to $Z_{A}$, we divide it into $D_{A}=D_{A_{1}}D_{A_{2}}\ldots D_{A_{\xi +1}}$, where $D_{A_{i}}$ represents the data in the kth rounds of the protocol, where k∈{i+n(ξ+1)∣ $n\in {\mathbb{N}}_{0},i+n\left(\xi +1\right)\leq N\}$. From the definitions, there is $Z_{A_{i}}\in D_{A_{i}}$, and, further, we can calculate that $\left({∁}_{Z_{A}}Z_{A_{i}}\right)\in \left({∁}_{D_{A}}D_{A_{i}}\right)$, where $\left({∁}_{D_{A}}D_{A_{i}}\right)$ represents the part of set $D_{A}$ excluding $D_{A_{i}}$, denoted as $D_{A_{1}}D_{A_{2}}\ldots D_{A_{i-1}}D_{A_{i+1}}\ldots D_{A_{\xi +1}}$. Using data processing inequality, we can calculate that $H\text{min}\epsilon_{i}{\left(Z_{A_{i}}|\left({∁}_{Z_{A}}Z_{A_{i}}\right)E^{\prime }\right)}_{\rho_{i}^{\prime }}\geq H_{\text{min}}^{\epsilon_{i}}{\left(Z_{A_{i}}|\left({∁}_{D_{A}}D_{A_{i}}\right)E^{\prime }\right)}_{\rho_{i}^{\prime }}$, where $\rho_{i}^{\prime }$ denotes the quantum state in the protocol that include the space $Z_{A_{i}}$, $\left({∁}_{D_{A}}D_{A_{i}}\right)$. Analyzing the entropy conditioned on ${∁}_{D_{A}}D_{A_{i}}$ rather than on ${∁}_{Z_{A}}Z_{A_{i}}$ allows us to work with a simpler quantum state based on pure states in the subsequent analysis, thereby facilitating the derivations that follow. The schematic diagram of the process is shown in Fig. 1, and, from this, we can give our first lemma.

Lemma 1. *The lower bound of the smooth min-entropy* $H_{\text{min}}^{\epsilon }{\left(Z_{A}|E^{\prime }\right)}_{\rho }$ *of an original protocol with a maximum correlation range* ξ *can be bound by the sum of the smooth min-entropies* $H_{\text{min}}^{\epsilon_{i}}{\left(Z_{A_{i}}|\left({∁}_{D_{A}}D_{A_{i}}\right)E^{\prime }\right)}_{\rho_{i}^{\prime }}$, *where each* $H_{\text{min}}^{\epsilon_{i}}{\left(Z_{A_{i}}|\left({∁}_{D_{A}}D_{A_{i}}\right)E^{\prime }\right)}_{\rho_{i}^{\prime }}$ *corresponds to the rounds in the original protocol that share the same modulo-*(ξ+1) *remainder, under the condition that all other rounds are made public. This relation satisfies*

$$\begin{array}{c} H_{\text{min}}^{\epsilon }{\left(Z_{A}|E^{\prime }\right)}_{\rho }\\ \geq \sum_{i=1}^{\xi +1}\left[H_{\text{min}}^{\epsilon_{i}}{\left(Z_{A_{i}}|\left({∁}_{D_{A}}D_{A_{i}}\right)E^{\prime }\right)}_{\rho_{i}^{\prime }}\right]-\sum_{i=1}^{\xi }\left(f_{i}\right) \end{array}$$ (1)

*where* $f_{1}=2{\text{log}}_{2}\frac{1}{\epsilon -2\epsilon_{1}-\epsilon_{1}^{\prime }}$, $f_{i}=2{\text{log}}_{2}\frac{1}{\epsilon_{i-1}^{\prime }-2\epsilon_{i}-\epsilon_{i}^{\prime }}$ *if* i∈[2,ξ], $\epsilon_{\xi +1}=\epsilon_{\xi }^{\prime }$.

*Proof.* See Materials and Methods for details.

Lemma 1 shows that the smooth min-entropy of a protocol with correlation can be bounded by the sum of the smooth min-entropies of its uncorrelated subcomponents. However, Lemma 1 cannot be directly used to compute a lower bound on the smooth min-entropy of the original protocol, because the subcomponents are required to be conditioned on the disclosure of the other parts. To address this, it is necessary to construct an uncorrelated protocol such that a certain part of it has the same smooth min-entropy as $H_{\text{min}}^{\epsilon_{i}}{\left(Z_{A_{i}}|\left({∁}_{D_{A}}D_{A_{i}}\right)E^{\prime }\right)}_{\rho_{i}^{\prime }}$, and, for this, we repeat the original protocol ξ+1 times to form a new protocol. Therefore, the new protocol actually executes (1+ξ)N rounds, which we refer to as physical rounds. We stipulate that the new protocol uses only kth physical rounds for the QKD process, which we call key generation rounds, where $k\in \{\left(i-1\right)N+i+n\left(\xi +1\right)|i\in \mathbb{N},n\in {\mathbb{N}}_{0},i+n\left(\xi +1\right)\leq N\}$, to keep the number of key generation rounds remain N, the same as the original protocol, while the left ξN physical rounds not only send the prepared quantum states through the channel but also publicly disclose all their data (all local ancillas or their measurement results, due to the requirements of the specific protocol), which we call leakage rounds. Similar to $Z_{A}$, the raw key bits of Alice in the new protocol, denoted as $Z_{A^{\prime }}$, can be categorized into ξ types and represented as $Z_{A}^{\prime }=Z_{A_{1}}^{\prime }Z_{A_{2}}^{\prime }\ldots Z_{A_{\xi +1}}^{\prime }$, where $Z_{A_{i}}^{\prime }$ represents the key sequence generated in the kth key generation round of the protocol, where $k\in \{\left(i-1\right)N+i+n\left(\xi +1\right)|n\in {\mathbb{N}}_{0},i+n\left(\xi +1)\leq N\right\}$. From the above discussion, we can also obtain that $Z_{A_{i}}$ and $Z_{A_{i}}^{\prime }$ correspond one to one. This correspondence is intuitively illustrated in Fig. 1, where the rounds of the original protocol are marked with the same color as the key generation rounds in the new protocol, while the leakage rounds are marked in white. For clarity, in summary, we give the definition of the new protocol.

Definition 1. *For any original protocol with a maximum correlation range* ξ, *we define a corresponding new protocol by repeating the original protocol* ξ+1 *times. In the* i*th repetition, only the rounds whose indices satisfy modulo* ξ+1 *congruent to* i *(with the remainder* ξ+1 *interpreted as* 0*) are used for key generation, while all other rounds are disclosed. The raw key of original protocol is denoted by* $Z_{A}$, *the raw key of the* i*th repetition of the new protocol is denoted by* $Z_{A_{i}}^{\prime }$, *and the raw key of the whole new protocol is denoted by* $Z_{A}^{\prime }$*. Similarly, define* $D_{A}^{\prime }=D_{A_{1}}^{\prime }D_{A_{2}}^{\prime }\ldots D_{A_{\xi +1}}^{\prime }$ *as the data from all disclosed rounds, where* $D_{A_{i}}^{\prime }$ *denotes the data revealed in the public rounds during the* i*th repetition of the original protocol.*

In the new protocol, all information $D_{A}^{\prime }$ is fully disclosed to treat these leakage rounds, together with their state preparation and auxiliary systems, as a side channel of the key generation rounds, thereby constructing the new protocol in which the local auxiliary systems contain only essential to the QKD process in the key generation rounds. Moreover, this construction is conducive to formulating a convenient pure state description for the analysis. In the new protocol, there exists an attack by Eve such that the joint density matrix of the raw key of the new protocol, the system of the leakage rounds and Eve’s system, satisfies ${⊗}_{i=1}^{\xi +1}\rho_{i}^{\prime \prime }$, where $\rho_{i}^{\prime \prime }$ is the density matrix of the rounds that generalize $Z_{A_{i}}^{\prime }$ and satisfies $\rho_{i}^{\prime \prime }=\rho_{i}^{\prime \prime }$. Thus we have $H_{\text{min}}^{{\tilde{\epsilon }}_{i}}{\left(Z_{A_{i}}|\left({∁}_{D_{A}}D_{A_{i}}\right)E^{\prime }\right)}_{\rho_{i}^{\prime }}=H_{\text{min}}^{{\tilde{\epsilon }}_{i}}{\left(Z_{A_{i}}^{\prime }|D_{A_{i}}^{\prime }E^{\prime }\right)}_{\rho_{i}^{\prime \prime }}$. Recall that the new protocol to publicly disclose all ancillas (or their measurement results) in leakage rounds and Eve’s optimal attack will also yield a smaller smooth min-entropy. Thus, we can calculate that $H_{\text{min}}^{{\tilde{\epsilon }}_{i}}{\left(Z_{A_{i}}^{\prime }|D_{A_{i}}^{\prime }E^{\prime }\right)}_{\rho_{i}^{\prime \prime }}\geq H_{\text{min}}^{{\tilde{\epsilon }}_{i}}{\left(Z_{A_{i}}^{\prime }|E^{\prime }\right)}_{\rho^{\prime }}$, where $\rho^{\prime }$ denotes the total quantum state of the raw key of the new protocol and Eve’s system. Thus, we give our second Lemma.

Lemma 2 *The lower bound of the smooth min-entropy* $H_{\text{min}}^{\epsilon }{\left(Z_{A}|E^{\prime }\right)}_{\rho }$ *of an original protocol with a maximum correlation range* ξ *can be bound by the sum of the smooth min-entropy* $H_{\text{min}}^{{\tilde{\epsilon }}_{i}}{\left(Z_{A_{i}}^{\prime }|E^{\prime }\right)}_{\rho^{\prime }}$ *of parts of the new protocol, where the definition of new protocol is in* *Definition 1**. This relation satisfies*

$$H_{\text{min}}^{\epsilon }{\left(Z_{A}|E^{\prime }\right)}_{\rho }\geq \sum_{i=1}^{\xi +1}\left[H_{\text{min}}^{\epsilon_{i}}{\left(Z_{A_{i}}^{\prime }|E^{\prime }\right)}_{\rho^{\prime }}\right]-\sum_{i=1}^{\xi }\left(f_{i}\right)$$ (2)

*where* $f_{1}=2{\text{log}}_{2}\frac{1}{\epsilon -2\epsilon_{1}-\epsilon_{1}^{\prime }}$, $f_{i}=2{\text{log}}_{2}\frac{1}{\epsilon_{i-1}^{\prime }-2\epsilon_{i}-\epsilon_{i}^{\prime }}$ *if* i∈[2,ξ], $\epsilon_{\xi +1}=\epsilon_{\xi }^{\prime }$.

*Proof.* See Materials and Methods for details.

Lemma 2 has already established a security constraint that connects a correlated protocol to an uncorrelated one. However, Lemma 2 requires estimating the smooth min-entropy of several subcomponents of the new protocol individually, which undoubtedly increases both the complexity and the impact of statistical fluctuations in the data. Therefore, a more effective approach is to further aggregate these parts and estimate them using the overall phase error rate. For most protocols, the smooth min-entropy can be estimated only for specific events, such as single-photon events in typical BB84 and MDI protocols, while multiphoton events cannot be directly estimated. Thus, it is necessary to apply the chain-rule result (*43*) once again to bound $H_{\text{min}}^{{\tilde{\epsilon }}_{i}}{\left(Z_{A_{i}}^{\prime }|E^{\prime }\right)}_{\rho^{\prime }}$, using $H_{\text{min}}^{{\tilde{\epsilon }}_{i}\prime }{\left(Z_{Z,A_{i}}^{\prime }|E^{\prime }\right)}_{\rho^{\prime }}$, where $Z_{A_{i}}^{\prime }=Z_{Z,A_{i}}^{\prime }Z_{X,A_{i}}^{\prime }$, in which $Z_{Z,A_{i}}^{\prime }$ denotes the parts that can estimated and $Z_{X,A_{i}}^{\prime }$ denotes the parts that cannot. Further, from the uncertainty relation and the parameter estimation of the QKD process (*51*), we can bound the $H_{\text{min}}^{{\tilde{\epsilon }}_{i}^{\prime }}{\left(Z_{Z,A_{i}}^{\prime }|E^{\prime }\right)}_{\rho^{\prime }}$ using $h\left({\bar{e_{i}}}_{\epsilon_{i}}^{U}\right)$, where ${\bar{e_{i}}}_{\epsilon_{i}}^{U}$ is the upper bound of the phase error rate $e_{i}$ and $h\left(x\right)=-x{\text{log}}_{2}\left(x\right)-$ $\left(1-x\right){\text{log}}_{2}\left(1-x\right)$. Because the smooth min-entropy is estimated using the phase error rate, its physical meaning lies in estimating based on a given phase error rate, with the failure probability bounded by the square of the smoothing parameter (*43*). Given the fact that, if each $Z_{Z,A_{i}}^{\prime }$ estimation fails, then the estimation of $Z_{Z,A}^{\prime }$ as a whole must also fail (and likewise for success), and, combining this with the convexity of h(x), we can use the total phase error rate and the overall failure probability to estimate the sum of the smooth min-entropies of each part. Consequently, together with Lemma 2, we can estimate the smooth min-entropy of the original protocol using the phase error rate of the newly constructed protocol, which gives us another lemma.

Proposition 1. *The lower bound of the smooth min-entropy* $H_{\text{min}}^{\epsilon }{\left(Z_{A}|E^{\prime }\right)}_{\rho }$ *of an original protocol with a maximum correlation range* ξ *can be bound by the upper bound of the estimation of phase error rate* ${\bar{e}}_{\hat{\epsilon }}^{U}$ *of the new protocol, where the definition of new protocol is in* *Definition 1*. *This relation satisfies*

$$H_{\text{min}}^{\epsilon }{\left(Z_{A}|E^{\prime }\right)}_{\rho }\geq n\left(1-h\left({\bar{e}}_{\hat{\epsilon }}^{U}\right)\right)-\xi f-\left(\xi +1\right)f^{\prime }$$ (3)

*where* ϵ*,* $\hat{\epsilon }$, *and* f *satisfy* $\hat{\epsilon }={\left(\frac{\epsilon -\xi \frac{1}{2^{f/2}}}{2\xi +1}-\frac{1}{2^{f^{\prime }/2}}\right)}^{\xi +1}$.

*Proof.* See Materials and Methods for details.

It is worth noting that, if all components of $Z_{A}$ can be used to estimate the phase error, then further scaling of $Z_{A_{i}}^{\prime }$ to $Z_{Z,A_{i}}^{\prime }$ is unnecessary. In this special case, we simply need to delete all terms containing $f^{\prime }$ from the conclusions in Eq. 3, which results in a more compact estimate of the smooth min-entropy.

Moreover, it is worth noting that, although our security proof requires a rearrangement of rounds as illustrated in Fig. 1, this rearrangement is not necessary in the actual data processing of the final protocol. Because the rounds are independent after the transformation, we are free to rearrangement them again to restore their original sequence.

### Secure QKD against correlated leakage source

After completing the security analysis framework, we will construct a secure QKD against correlated leakage source based on the two-state SNS protocol (*28*–*31*). To analyze the security, we impose a lower bound constraint on the vacuum component of the state sent by the source into the channel (*28*–*31*). The same constraint can be achieved through precharacterization, limiting the upper bounds of pulse intensities or other methods, and it has already been experimentally implemented (*33*). The specific assumption is as follows.

Assumption 2. *For the two-state SNS-QKD protocol, the lower bound of the proportion of vacuum states in each round, under both the send and not-send scenarios, is known. Specifically, given the* i*th round and its preceding* ξ *rounds, the state sent into the channel during the current round* $\rho_{r_{i-\xi }^{i},a_{i-\xi }^{i}}^{A\left(B\right)}$ *satisfies*

$$\underset{r_{i-\xi }^{i-1}}{\text{min}}\left(\underset{a_{i-\xi }^{i}}{\text{min}}\left(|\langle 0|\rho_{r_{i-\xi }^{i},a_{i-\xi }^{i}}^{A\left(B\right)}|0\rangle |\right)\right)\geq V_{r_{i}}^{A\left(B\right)}$$ (4)

*where* $r_{i}\in \left\{0,1\right\}$ *denotes the encoding setting in* i*th round;* $a_{i}$ *denotes the set of ancillas in the system that are potentially related to rounds and can influence the transmitted state, which includes controls over SPF, correlation, side channel, and so on;* $V_{r_{i}}^{A\left(B\right)}$ *denotes the lower bound of the proportion of vacuum states; and the sequence from* i*th to the* j*th round for* a *and* r *is defined as* $a_{i}^{j}≔a_{j}a_{j-1}\ldots a_{i}$ *and* $r_{i}^{j}≔r_{j}r_{j-1}\ldots r_{i}$, *respectively.*

Following the above approach, we construct an equivalent protocol. Furthermore, by applying a unitary mapping to the transmitted states, we establish the security equivalence between the equivalent protocol and an i.i.d. protocol. On the basis of this, we can estimate the secret key rate.

**Fig. 2.**
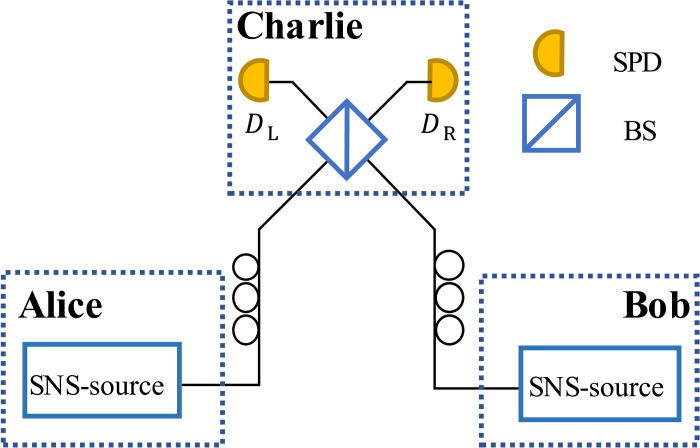
The schematic diagram of the protocol. $D_{L}$ and $D_{R}$ represent the left and right detectors, respectively. SPD, single-photon detector; BS, 50:50 beam splitter; SNS-source, a system comprising a weak coherent source and modulation devices, satisfying Assumptions 1 and 2.

Before introducing the protocol, we first outline some fundamental requirements and definitions for its implementation. The protocol involves two users, Alice and Bob, as well as an untrusted node, Charlie. In each round, Alice and Bob attempt to prepare either a coherent state with a known intensity or a vacuum state, following the sending-or-not-sending strategy (*14*, *28*–*31*). However, due to the limitations of their sources, they can only generate quantum states that satisfy Assumptions 1 and 2. For the untrusted node Charlie, if honest, she performs an interference measurement shown in Fig. 2. Additionally, she must compensate for channel fluctuations so that constructive interference occurs at the left detector and destructive interference at the right detector. After all transmissions and measurements are completed, Alice and Bob negotiate to select a subset of rounds with probability $p_{\text{PE}}$, marked as parameter estimation rounds, for parameter estimation, while the remaining rounds are marked as key extraction rounds. Furthermore, we need to define certain events, a Z event occurs when exactly one of Alice or Bob chooses $r_{i}=0$, an O event occurs when both Alice and Bob choose $r_{i}=0$, and a B event occurs when both Alice and Bob choose $r_{i}=1$. Based on these definitions, our protocol proceeds as follows.

1) State preparation: In the ith round (i=1,2,…,N), Alice and Bob each independently choose a bit setting $r_{i}\in R=\left\{0,1\right\}$. Then, on the basis of the value of $r_{i}$, they send the prepared quantum state with a known lower bound on its vacuum component (satisfying Assumptions 1 and 2) into the quantum channel, where they will be measured by an untrusted nod Charlie.

2) Measurement: If honest, the untrusted nod Charlie performs an interference measurement on the states sent by Alice and Bob in the ith round. If only the right detector clicks, Charlie records this event as a successful measurement. Charlie public whether each round is successful.

3) Parameter estimation: Through classical communication, Alice and Bob confirm the number of Z, O, and B events, denoted as $n_{Z}^{\text{PE}}$, $n_{O}^{\text{PE}}$, and $n_{B}^{\text{PE}}$, among all successful and parameter estimation rounds. They then determine the quantum bit error rate $e_{\text{bit}}$ and estimate the upper bound of phase error rate ${\bar{n}}_{\text{ph}}$ and the lower bound of the number of Z ${\underline{n}}_{Z}$ in the key extraction rounds (using method below).

4) Key distillation: Alice and Bob perform error correction and privacy amplification based on the results of the parameter estimation step and then use the data in the successful rounds to generate the secret keys.

We consider the entanglement-equivalent protocol for N rounds. If there is an ideal source, then the protocol can be write as

$${|\Phi \rangle }^{\text{ide}}={|\Phi \rangle }_{A}^{\text{ide}}⊗{|\Phi \rangle }_{B}^{\text{ide}}⊗{|\Phi \rangle }_{\text{PE}}$$ (5)

where ${|\Phi \rangle }_{\text{PE}}=\left[\sum_{m_{1}^{N}}\left(\prod_{i=1}^{N}\sqrt{p_{m_{i}}^{\text{PE}}}\right)\left({⊗}_{i=1}^{N}{|m_{i}\rangle }_{{\text{PE}}_{i}}\right)\right]$ denotes a set of auxiliaries used to determine whether a given round is selected for parameter estimation, $m_{i}=1$ indicates that the ith round is used for parameter estimation, while $m_{i}=0$ means it is not, and $p_{1}^{\text{PE}}=1-p_{0}^{\text{PE}}=p_{\text{PE}}$

$${|\Phi \rangle }_{A}^{\text{ide}}=\left[\sum_{r_{1}^{N}}\left(\prod_{i=1}^{N}\sqrt{p_{r_{i}}}\right)\left(\overset{N}{\underset{i=1}{⊗}}{|r_{i}\rangle }_{A_{i}}{|\psi_{r_{i}}^{\text{ide}}\rangle }_{C_{i}}\right)\right]$$ (6)

where $p_{r_{i}}$ is the probability of selecting $r_{i}$, ${|\psi_{r_{i}}^{\text{ide}}\rangle }_{C_{i}}$ denotes the ideal encoded coherent state with selected state $r_{i}$ to be send into channel, and ${|\Phi \rangle }_{B}^{\text{ide}}$ is similar to Eq. 6. However, in the following discussion, we omit ${|\Phi \rangle }_{\text{PE}}$, as it is an auxiliary tag negotiated by Alice and Bob after state transmission that introduces no imperfections and is tensor producted with the remaining transmitted part; therefore, all transformations do not involve this component. For the protocol is ideal, we have that ${|\psi_{0}^{\text{ide}}\rangle }_{C_{i}}=|0\rangle$ and ${|\psi_{1}^{\text{ide}}\rangle }_{C_{i}}=|\mu \rangle$, where ∣0〉 and ∣μ〉 denote the ideal coherent state without any imperfections. Protocols like those in Eq. 6 have been proved secure (*28*–*31*); however, if the source is imperfect, then the form will be more complex. As we have discussed in Assumption 2, instead of ${|\psi_{r_{i}}^{\text{ide}}\rangle }_{C_{i}}$, Alice’s source send $\rho_{r_{i-\xi }^{i},a_{i-\xi }^{i}}^{A}$. Consider a protocol that sends the purification ${|\psi_{r_{i-\xi }^{i},a_{i-\xi }^{i}}^{\text{imp}}\rangle }_{C_{i}}$ of $\rho_{r_{i-\xi }^{i},a_{i-\xi }^{i}}^{A}$ into the channel, the security of this protocol can ensure the security of the protocol that sends $\rho_{r_{i-\xi }^{i},a_{i-\xi }^{i}}^{A}$. Thus, if the source is imperfect, then the entanglement-equivalent protocol of Alice’s side Eq. 6 will become

$${|\Phi \rangle }_{A}=[\sum_{r_{1}^{N}a_{1}^{N}}\left(\prod_{i=1}^{N}\sqrt{p_{r_{i}}q_{a_{i}}}\right)\left(\overset{N}{\underset{i=1}{⊗}}{|r_{i}\rangle }_{A_{i}}{|a_{i}\rangle }_{A_{i}^{\prime \prime }}{|\psi_{r_{i-\xi }^{i},a_{i-\xi }^{i}}^{\text{imp}}\rangle }_{C_{i}}\right)]$$ (7)

where $q_{a_{i}}$ is the probability of selecting $a_{i}$.

Treat the protocol in Eq. 7 as the original protocol described in the “Security analysis framework addressing correlated sources” section, and, then, the new protocol without correlation can be expressed as

$$\begin{array}{c} {|\Phi \rangle }_{A}^{\text{new}}=\left[\sum_{{r^{\prime }}_{1}^{\xi N}}\sum_{a_{1}^{\left(1+\xi \right)N}}\right.\\ \left(\prod_{i=1}^{\xi N}\sqrt{p_{r_{i}^{\prime }}}\prod_{j=1}^{\left(1+\xi \right)N}\sqrt{q_{a_{j}}}\right)\left(\overset{\xi N}{\underset{i=1}{⊗}}{|r_{i}^{\prime }\rangle }_{A_{i}^{\prime }}\overset{\left(1+\xi \right)N}{\underset{j=1}{⊗}}{|a_{j}\rangle }_{A_{j}^{\prime \prime }}\right)\\ \left.⊗\left[\sum_{r_{1}^{N}}\left(\prod_{i=1}^{N}\sqrt{p_{r_{i}}}\right)\left(\overset{N}{\underset{i=1}{⊗}}{|r_{i}\rangle }_{A_{i}}{|\psi_{r_{i},r^{\prime }\left(i,\xi \right),a\left(i,\xi \right)}^{{\text{imp}}^{\prime }}\rangle }_{C_{i}^{\prime }}\right)\right]\right] \end{array}$$ (8)

where $r^{\prime }\left(i,\xi \right)$ denotes the local ancilla in the previous ξ physical rounds and the following ξ physical rounds of the ith key generation round, a(i,ξ) denotes the system ancilla of the up mentioned physical rounds and the ith key generation round, and ${|\psi_{r_{i},r^{\prime }\left(i,\xi \right),a\left(i,\xi \right)}^{{\text{imp}}^{\prime }}\rangle }_{C_{i^{\prime }}}$ denotes the state sent into the channel in the ith key generation round and the following ξ physical rounds (detailed in Materials and Methods and Supplementary Text).

As we have discussed, the new protocol reveals all physical rounds except the key generation rounds. Furthermore, because the security of the original protocol is constrained by that of the new protocol, we can relax the assumptions on the new protocol. Therefore, we further disclose the ancilla in the space $A_{i}^{\prime \prime }$ for all physical rounds and assume that Alice sends additional quantum states into the channel. Thus, for any additional state ${|\psi_{r_{i},r^{\prime }\left(i,\xi \right),a\left(i,\xi \right)}^{\text{add}}\rangle }_{C_{i}^{\prime \prime }}$ sent into the channel, we have a protocol ${|\Phi \rangle }_{A}^{{\text{new}}_{2}}$ that, based on ${|\Phi \rangle }_{A}^{\text{new}}$ sending ${|\psi_{r_{i},r^{\prime }\left(i,\xi \right),a\left(i,\xi \right)}^{{\text{imp}}^{\prime }}\rangle }_{C_{i}^{\prime }}$ in Eq. 8, instead sends ${|\psi_{r_{i},r^{\prime }\left(i,\xi \right),a\left(i,\xi \right)}^{{\text{imp}}^{\prime }}\rangle }_{C_{i}^{\prime }}{|\psi_{r_{i},r^{\prime }\left(i,\xi \right),a\left(i,\xi \right)}^{\text{add}}\rangle }_{C_{i}^{\prime \prime }}$. Then, the security of protocol ${|\Phi \rangle }_{A}^{\text{new}}$ in Eq. 8 can be guaranteed by protocol ${|\Phi \rangle }_{A}^{{\text{new}}_{2}}$.

Further, we can prove that there exist a set of additional state ${|\psi_{r_{i},r^{\prime }\left(i,\xi \right),a\left(i,\xi \right)}^{\text{add}}\rangle }_{C_{i}^{\prime \prime }}$ and a unitary mapping U acting in space ${⊗}_{i=1}^{\mathrm{\xi N}}A_{i}^{\prime }{⊗}_{j=1}^{\left(1+\xi \right)N}A_{j}^{\prime \prime }{⊗}_{i=1}^{N}C_{i}^{\prime }C_{i}^{\prime \prime }$ that makes protocol ${|\Phi \rangle }_{A}^{{\text{new}}_{2}}$ become an equivalent protocol ${|\Phi \rangle }_{A}^{\text{equ}}$, which is i.i.d. and $\left({⊗}_{i=1}^{\xi N}{|r_{i}^{\prime }\rangle }_{A_{i^{\prime }}}{⊗}_{j=1}^{\left(1+\xi \right)N}{|a_{j}\rangle }_{A_{j}^{\prime \prime }}\right)$ no longer appears entangled within the protocol but instead appears in a tensor product form. Thus, because $r^{\prime }$ and a no longer play any role, we simplify the equivalent protocol ${|\Phi \rangle }_{A}^{\text{equ}}$ by removing them. The final equivalent protocol then satisfies

$${|\Phi \rangle }_{A}^{\text{equ}}=\left[\sum_{r_{1}^{N}}\left(\prod_{i=1}^{N}\sqrt{p_{r_{i}}}\right)\left(\overset{N}{\underset{i=1}{⊗}}{|r_{i}\rangle }_{A_{i}}{|\psi_{r_{i}}^{\text{equ}}\rangle }_{C_{i}^{\prime \prime \prime }}\right)\right]$$ (9)

where ${|\psi_{0}^{\text{equ}}\rangle }_{C_{i}^{\prime \prime \prime }}=|0\rangle$ and ${|\psi_{1}^{\text{equ}}\rangle }_{C_{i}^{\prime \prime \prime }}=|\mu_{\text{equ}}\rangle$, where ∣0〉 is the vacuum state and $|\mu_{\text{equ}}\rangle$ is the coherent state with an average number of photons equals to $\mu_{\text{equ}}$, satisfies$e^{-\mu_{\text{equ}}}={\left[\sqrt{V_{0}^{A,\xi }V_{1}^{A,\xi }}-\sqrt{\left(1-V_{0}^{A,\xi }\right)\left(1-V_{1}^{A,\xi }\right)}\right]}^{2}$ and $V_{r_{i}}^{A,\xi }=V_{r_{i}}^{A}{\left(p_{0}\sqrt{V_{0}^{A}}+p_{1}\sqrt{V_{1}^{A}}\right)}^{2\xi }$ (detailed in Materials and Methods and Supplementary Text).

The above analysis is also applicable to Bob’s side. In this case, we can conclude that a two-state SNS-QKD protocol satisfying Assumptions 1 and 2, including the presence of SPFs, side channels, and correlations, has its minimum smooth entropy constrained by the phase error rate of the equivalent protocol under the same measurement outcomes. In addition, the equivalent protocol ultimately satisfies

$$\begin{array}{c} {|\Phi \rangle }^{\text{equ}}=\left[\sum_{r_{1}^{N}}\left(\prod_{i=1}^{N}\sqrt{p_{r_{i}}}\right)\left(\overset{N}{\underset{i=1}{⊗}}{|r_{i}\rangle }_{A_{i}}{|\psi_{r_{i}}^{\text{Aequ}}\rangle }_{C_{i}^{A}}\right)\right]\\ ⊗\left[\sum_{r_{1}^{N}}\left(\prod_{i=1}^{N}\sqrt{p_{r_{i}}}\right)\left(\overset{N}{\underset{i=1}{⊗}}{|r_{i}\rangle }_{B_{i}}{|\psi_{r_{i}}^{\text{Bequ}}\rangle }_{C_{i}^{B}}\right)\right]⊗{|\Phi \rangle }_{\text{PE}} \end{array}$$ (10)

where ${|\psi_{0}^{A\left(B\right)\text{equ}}\rangle }_{C_{i}^{A\left(B\right)}}=|0\rangle$, ${|\psi_{1}^{A\left(B\right)\text{equ}}\rangle }_{C_{i}^{A\left(B\right)}}=|\mu_{\text{equ}}^{A\left(B\right)}\rangle$, and $e^{-\mu_{\text{equ}}^{A\left(B\right)}}={\left[\sqrt{V_{0}^{A\left(B\right),\xi }V_{1}^{A\left(B\right),\xi }}-\sqrt{\left(1-V_{0}^{A\left(B\right),\xi }\right)\left(1-V_{1}^{A\left(B\right),\xi }\right)}\right]}^{2}$, $V_{r_{i}}^{A\left(B\right),\xi }=V_{r_{i}}^{A\left(B\right)}{\left(p_{0}\sqrt{V_{0}^{A\left(B\right)}}+p_{1}\sqrt{V_{1}^{A\left(B\right)}}\right)}^{2\xi }$, and, at this step, we recall the auxiliary particle ${|\Phi \rangle }_{\text{PE}}$, which indicates whether the rounds are selected for parameter estimation.

After completing the above analysis, from Proposition 1, we still need to estimate the phase error rate of the equivalent protocol ${|\Phi \rangle }^{\text{equ}}$ to derive our final key rate formula. Now, the security of protocols similar to that in Eq. 10 has already been proven within the framework of SCS QKD (*28*–*31*, *52*). We choose to use postselection security analysis (*31*, *53*). For a two-state SNS QKD described in Eq. 10, under collective attack, we can calculate the upper bound of phase error probability $P_{\text{ph}}$, satisfies

$$\begin{array}{c} P_{\text{ph}}\leq \frac{p_{1}p_{0}}{2}\left(c_{0}^{2}\frac{P_{O}}{{\left(p_{0}\right)}^{2}}+c_{1}^{2}\frac{P_{B}}{{\left(p_{1}\right)}^{2}}+{\bar{c}}_{2}^{2}\right.\\ \left.+2c_{0}c_{1}\sqrt{\frac{P_{O}P_{B}}{{\left(p_{0}\right)}^{2}{\left(p_{1}\right)}^{2}}}+c_{0}{\bar{c}}_{2}\sqrt{\frac{P_{O}}{{\left(p_{0}\right)}^{2}}}+c_{1}{\bar{c}}_{2}\sqrt{\frac{P_{B}}{{\left(p_{1}\right)}^{2}}}\right) \end{array}$$ (11)

where $P_{O}$ and $P_{B}$ are the probabilities of the O event and the B event of key extraction rounds (detailed in Materials and Methods).

Noting that obtaining Eq. 11 requires the assumption that Eve’s attack is a collective attack, we can conclude that the measurement outcome probabilities are identical for all rounds. Moreover, we cannot directly obtain the number $n_{O}$ and $n_{B}$ of the O events and the B events in the key extraction rounds; instead, we can only calculate $n_{O}^{\text{PE}}$ and $n_{B}^{\text{PE}}$. In addition, because of Eve performs a collective attack, $P_{O}/\left(1-p_{\text{PE}}\right)$ and $P_{B}/\left(1-p_{\text{PE}}\right)$ are equal to the corresponding portions from the parameter estimation rounds, which are denoted as $P_{O}^{\text{PE}}/p_{\text{PE}}$ and $P_{B}^{\text{PE}}/p_{\text{PE}}$. Therefore, using the Chernoff bound (*54*, *55*), we can separately estimate their upper bounds such that $P_{O}=\left(\left(1-p_{\text{PE}}\right)/p_{\text{PE}}\right)P_{O}^{\text{PE}}\leq \left(\left(1-p_{\text{PE}}\right)/p_{\text{PE}}\right)\;\bar{\text{Cher}}\;\left(n_{O}^{\text{PE}},\epsilon_{\text{ph}}\right)/N$, $P_{B}=\left(\left(1-p_{\text{PE}}\right)/p_{\text{PE}}\right)P_{B}^{\text{PE}}\leq P_{B}=\left(\left(1-p_{\text{PE}}\right)/p_{\text{PE}}\right)P_{B}^{\text{PE}}\leq \left(\left(1-p_{\text{PE}}\right)/p_{\text{PE}}\right)\bar{\text{Cher}}\left(n_{B}^{\text{PE}},\epsilon_{\text{ph}}\right)/N$, where $\bar{\text{Cher}}\left(\cdot ,\epsilon \right)$ is the upper bound of the expectation estimated from the observation with a failure probability ϵ (*31*) and satisfies $\bar{\text{Cher}}\left(X,\epsilon_{x}\right)=X+\text{ln}\frac{1}{\epsilon_{x}}+\sqrt{{\text{ln}}^{2}\frac{1}{\epsilon_{x}}+2X\text{ln}\frac{1}{\epsilon_{x}}}$, $\underline{\text{Cher}}\left(X,\epsilon_{x}\right)=X+\frac{1}{2}\text{ln}\frac{1}{\epsilon_{x}}-\frac{1}{2}\sqrt{{\text{ln}}^{2}\frac{1}{\epsilon_{x}}+8X\text{ln}\frac{1}{\epsilon_{x}}}$.

Consequently, the phase error number satisfies $n_{\text{ph}}\leq {\bar{n}}_{\text{ph}}=$ $\bar{\text{cher}}\left({\mathrm{NP}}_{\text{ph}},\epsilon_{\text{ph}}\right)$, where $\bar{\text{cher}}\left(\cdot ,\epsilon \right)$ is the upper bound of the observation estimated from the expectation with a failure probability ϵ (*31*) and satisfies $\bar{\text{cher}}\left(E,\epsilon_{x}\right)=E+\frac{1}{2}\text{ln}\frac{1}{\epsilon_{x}}+\frac{1}{2}\sqrt{{\text{ln}}^{2}\frac{1}{\epsilon_{x}}+8E\text{ln}\frac{1}{\epsilon_{x}}}$, $\underline{\text{cher}}\left(E,\epsilon_{x}\right)=$ $E-\sqrt{2E\text{ln}\frac{1}{\epsilon_{x}}}$. Then, by using the de Finetti reduction with fixed marginal (*56*), we can extend the phase error estimation to the case of Eve’s coherent attacks by appropriately increasing the failure probability of the estimation of ${\bar{n}}_{\text{ph}}$ (*31*). Specifically, in the case of Eve’s coherent attacks, the upper bound of the phase error with the failure probability $\epsilon_{\text{ph}}$ satisfies

$${\bar{n}}_{\text{ph}}^{c,\epsilon_{\text{ph}}}=\bar{\text{cher}}\left({\mathrm{NP}}_{\text{ph}},\frac{\epsilon_{\text{ph}}}{3g_{N,64}}\right)$$ (12)

where $g_{N,x}=\left(\begin{array}{c} N+x-1\\ N \end{array}\right)\leq {\left(\frac{e\left(N+x-1\right)}{x-1}\right)}^{x-1}$ (*56*), and x is the square of the dimension of Alice, Bob, and Charlie, which here we choose $x=d_{A}^{2}d_{B}^{2}d_{C}^{2}d_{\text{PE}}^{2}=2^{2}\times 2^{2}\times 2^{2}\times 2^{2}=256$ (*31*).

Moreover, we cannot directly measure the number $n_{Z}$ of Z events and in the key extraction rounds. Instead, we estimate them using the corresponding occurrences in the parameter estimation rounds. By applying the Chernoff bound (*54*, *55*), we can calculate $p_{\text{PE}}\left(n_{Z}+n_{Z}^{\text{PE}}\right)\geq \underline{\text{Cher}}\left(n_{Z}^{\text{PE}},\epsilon_{Z}\right)$. Thus, the lower bound of $n_{Z}$ with failure probability $\epsilon_{Z}$ satisfies

$${\underline{n}}_{Z}^{\epsilon_{Z}}=\frac{\underline{\text{Cher}}\left(n_{Z}^{\text{PE}},\epsilon_{Z}\right)}{p_{\text{PE}}}-n_{Z}^{\text{PE}}$$ (13)

Recall the original protocol, because of the property of the two-universal hash function (*57*), if the secure key have a length l with $\epsilon_{\text{tot}}$ secure, then $l=H_{\text{min}}^{\epsilon }\left(Z|E\right)-2{\text{log}}_{2}\frac{1}{2\bar{\epsilon }}$ with $\epsilon_{\text{tot}}=2\epsilon +\bar{\epsilon }$. Next, consider the error correction process. Suppose ${\mathrm{fn}}_{Z}h\left(e_{\text{bit}}\right)$ classical bits are consumed during error correction, where f is the efficiency of the error correction. If the key is $\epsilon_{\text{cor}}$ correct, then a hash with length ${\text{log}}_{2}\frac{2}{\epsilon_{\text{cor}}}$ must be announced for error correction. Then, the key length become

$$l=H_{\text{min}}^{\epsilon }\left(Z|E^{\prime }\right)-{\mathrm{fn}}_{Z}h\left(e_{\text{bit}}\right)-{\text{log}}_{2}\frac{2}{\epsilon_{\text{cor}}}-2{\text{log}}_{2}\frac{1}{2\bar{\epsilon }}$$ (14)

where $\epsilon_{\text{tot}}=2\epsilon +\bar{\epsilon }+\epsilon_{\text{cor}}$ and E is Eve’s system before the error correction (*51*).

Due to the conclusion in Eq. 14, the final key rate length is transformed into an estimation problem for the smooth min-entropy of Z in the original protocol. We can apply the conclusions from Proposition 1 to convert this problem into an estimation of phase errors in the equivalent protocol, completing the key rate estimation. From Eqs. 12 and 13 and according to Eq. 3, we can obtain that

$$\begin{array}{c} H_{\text{min}}^{\epsilon }\left(Z|E^{\prime }\right)\geq {\underline{n}}_{Z}^{\epsilon_{0}}\left(1-h\left(\frac{{\bar{n}}_{\text{ph}}^{c,{\left(\epsilon_{1}\right)}^{2}}}{{\underline{n}}_{Z}^{\epsilon_{0}}}\right)\right)-2\xi {\text{log}}_{2}\frac{1}{\epsilon_{2}}\\ -2\left(\xi +1\right){\text{log}}_{2}\frac{1}{\epsilon_{3}} \end{array}$$ (15)

then we can calculate the key length $l_{\text{max}}$ that satisfies

$$\begin{array}{c} l_{\text{max}}={\underline{n}}_{Z}^{\epsilon_{0}}\left(1-h\left(\frac{{\bar{n}}_{\text{ph}}^{c,{\left(\epsilon_{1}\right)}^{2}}}{{\underline{n}}_{Z}^{\epsilon_{0}}}\right)\right)-{\mathrm{fn}}_{Z}h\left(e_{\text{bit}}\right)-{\text{log}}_{2}\frac{2}{\epsilon_{\text{cor}}}\\ -2{\text{log}}_{2}\frac{1}{2\bar{\epsilon }}-2\xi {\text{log}}_{2}\frac{1}{\epsilon_{2}}-2\left(\xi +1\right){\text{log}}_{2}\frac{1}{\epsilon_{3}} \end{array}$$ (16)

where ${\bar{n}}_{\text{ph}}^{c,{\left(\epsilon_{1}\right)}^{2}}$ satisfies Eq. 12, ${\underline{n}}_{Z}^{\epsilon_{0}}$ satisfies Eq. 13, and $\epsilon_{\text{tot}}=2\epsilon +$ $\bar{\epsilon }+\epsilon_{\text{cor}}+\epsilon_{0}$ and $\epsilon_{1}={\left(\frac{\epsilon -{\xi \epsilon }_{2}}{2\xi +1}-\epsilon_{3}\right)}^{\xi +1}$.

### Simulation result of correlated leakage source secure QKD

Through simulations, we can verify the performance of our protocol. Specifically, we set the misalignment error rate to 1%, the detector dark count rate to $p_{d}=10^{-9}$ bit per pulse, the error correction efficiency to f=1.16, the extinction ratio between sending and nonsending intensities to 1/1000 (*45*, *46*), and the security parameter to $\epsilon_{\text{tot}}=10^{-10}$. For the specific security parameters, we set $\epsilon =\bar{\epsilon }=\epsilon_{\text{cor}}=\epsilon_{0}=\epsilon_{\text{tot}}/5$ and $\epsilon_{2}=\epsilon_{3}=\epsilon^{2}$. Further, we assume that the attenuation from Alice and Bob to the interference node is identical, and they have the same upper bound of the intensity, the range of correlations, as well as the sending probabilities. By optimizing the sending probability and upper bound of intensity, we obtain the attenuation-key rate curves for different correlation ranges ξ.

**Fig. 3.**
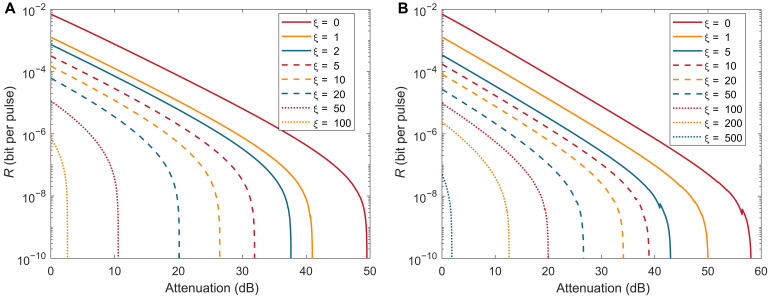
The simulation result of the performance of our protocol. We set the misalignment error rate to 1%, detector dark count rate to $p_{d}=10^{-9}$ bit per pulse, the error correction efficiency to f=1.16, the extinction ratio between sending and nonsending intensities to 1/1000, and the security parameter to $\epsilon_{\text{tot}}=10^{-10}$. (**A**) Secret key rate when $N=10^{12}$. (**B**) Secret key rate when $N=10^{14}$.

**Fig. 4.**
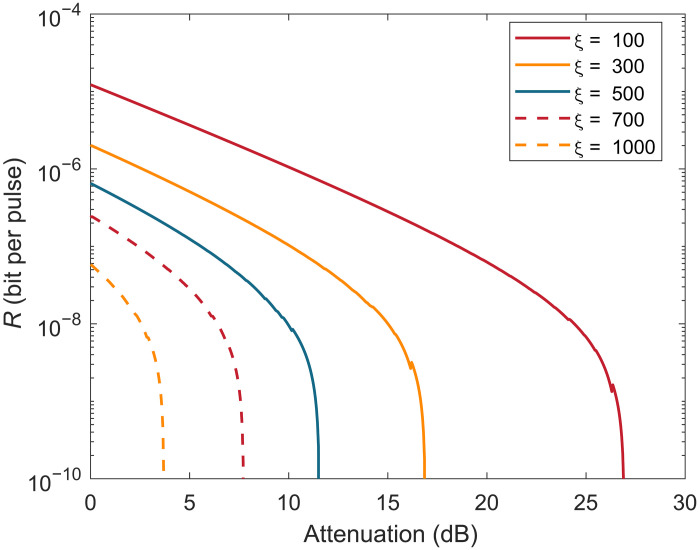
The simulation result of the performance of our protocol when $N=2\times 10^{15}$. We set the misalignment error rate to 1%, detector dark count rate to $p_{d}=10^{-9}$ bit per pulse, the error correction efficiency to f=1.16, the extinction ratio between sending and nonsending intensities to 1/1000, and the security parameter to $\epsilon_{\text{tot}}=10^{-10}$.

Figures 3 and 4 show the numerical simulations results of our protocol. In Fig. 3, we consider two common scenarios where the total number of pulses sent by Alice or Bob, N, which leads to the finite-length effect, is set to 10^12^ and 10^14^, corresponding to Fig. 3 (A and B), respectively. In both cases, we observe that, when there is no correlation (i.e., ξ=0), our protocol, according to the analysis in our manuscript, reduces to the existing SCS-QKD protocol, and the simulation results align with those of SCS-QKD. Additionally, we find that the smaller the correlation range ξ, the greater its impact on the key rate and the maximal transmission attenuation. For $N=10^{12}$, increasing the correlation range from ξ=0 to 1 results in a maximum attenuation loss of about 9 dB, while increasing the correlation range from ξ=1 to 5 also leads to an additional 9 dB too. Furthermore, in the cases of $N=10^{12}$ and 10^14^, we simulate correlation ranges up to ξ=100 and 500, respectively. In Fig. 4, we consider a scenario with a larger N. By setting $N=2\times 10^{15}$, we find that our protocol can still generate keys even when the correlation range is very large, reaching up to ξ=1000.

## DISCUSSION

We have addressed a critical security challenge in practical QKD with correlated leakage source. We first proposed a security analysis framework that allows for finite-key analysis in the presence of correlations by extending and rearranging QKD rounds and applying the generalized chain rule. Furthermore, we introduced a secure QKD against correlated leakage source, extending SCS QKD to scenarios where SPFs, side channels, and correlations coexist. By characterizing the correlation range ξ and imposing a lower bound on the vacuum component of the transmitted states of the correlated leakage source, we effectively address the security loopholes introduced by source imperfections.

Unlike existing approaches, our protocol does not require explicit characterization or magnitudes of correlation and side channel, making it more practical for real-world QKD systems. Our simulation results demonstrate the protocol’s strong robustness, effectiveness, and reliability, especially against very high order correlations. From the simulation results, we can see that our protocol substantially improves tolerance to correlation range compared to existing protocols. Most existing protocols require prior knowledge of not only the correlation range but also the further characterization of both correlation and side channel before conducting security analysis (*17*, *36*–*41*). Even for very small correlation levels, the analyzable correlation range typically does not exceed ξ=10. Moreover, existing experiments that measure correlation generally suggest that the correlation range ξ is primarily concentrated between 1 and 3 (*36*, *41*, *45*–*50*), with the worst case scenarios reaching only up to ξ=6 (*44*). Simulation results show that our protocol can handle a correlation range far beyond that of existing protocols and can encompass all now measured correlation parameters of real devices and systems. Under practical device parameters (*36*, *41*, *44*–*50*, *58*, *59*), most existing protocols struggle to generate secure keys, with only a few achieving this through correlation suppression, special encoding structures, or source monitor module (*41*). In contrast, our protocol only requires characterization of the correlation range without the need for complex additional steps. Furthermore, current correlation analysis protocols have not yet incorporated finite-length effects, whereas our protocol is the first to achieve this. Compared to existing solutions, our framework and protocol substantially enhance the security and feasibility of QKD under realistic device imperfections.

To avoid explicitly characterizing the magnitude and specific form of the SPF, side channel, and correlation, our protocol deliberately incurs the cost of a linear secret key rate scaling. While the underlying security analysis framework is, in principle, extensible to protocols such as MP QKD and TF QKD (*12*–*16*), surpassing the linear bound would require additional characterization of correlations across multiple source degrees of freedom tailored for those protocols. In addition, it seems that there are few experimental works on the source characterization for MP QKD and TF QKD, as far as we know. Therefore, retaining a linear key-rate scaling (not covering MP QKD and TF QKD) represents a reasonable and necessary trade-off at the present stage.

However, once certain improvements are made to the existing characterization methods for characterizing correlation and side channel (*36*, *41*, *44*–*50*) (or example, by providing a complete and simultaneous quantification of the correlation magnitudes in the intensity, phase, and state preparation degrees), our framework also holds the potential to address such issues beyond the linear bound. Moreover, once characterization techniques are incorporated, both our protocol and prospective improved variants for TF QKD can be implemented under current experimental conditions, enabling a final treatment of correlation problems.

As modern society’s demand for data security continues to grow, ensuring the secure deployment of QKD has become an urgent challenge. By adopting our security analysis framework, the final security barrier, correlations, can now be effectively addressed under finite-key conditions. Furthermore, with our correlated leakage source secure protocol, all security loopholes in QKD can be closed through a simple characterization of the source, paving the way for practical and truly secure QKD implementations. This represents an important advancement toward closing security loopholes in QKD and achieving high-performance, real-world implementations. Future research could further explore optimizing key rates under correlated conditions and extending the framework to other QKD protocols to enhance their practical security.

## MATERIALS AND METHODS

### Proof of Lemma 1 and 2

Recall the definitions in the “Security analysis framework addressing correlated sources” section in Results; by the generalized chain-rule result (*43*) and data processing inequality, we can obtain that

$$\begin{array}{c} H_{\text{min}}^{\epsilon }{\left(Z_{A}|E^{\prime }\right)}_{\rho }=H_{\text{min}}^{\epsilon }{\left(Z_{A_{1}}Z_{A_{2}}\ldots Z_{A_{\xi +1}}|E^{\prime }\right)}_{\rho }\\ \geq H_{\text{min}}^{\epsilon_{1}}{\left(Z_{A_{1}}|Z_{A_{2}}Z_{A_{3}}\ldots Z_{A_{\xi +1}}E^{\prime }\right)}_{\rho }\\ +H_{\text{min}}^{\epsilon_{1}^{\prime }}{\left(Z_{A_{2}}\ldots Z_{A_{\xi +1}}|E^{\prime }\right)}_{\rho }-f_{1}\\ \geq H_{\text{min}}^{\epsilon_{1}}{\left(Z_{A_{1}}|Z_{A_{2}}Z_{A_{3}}\ldots Z_{A_{\xi +1}}E^{\prime }\right)}_{\rho }\\ +H_{\text{min}}^{\epsilon_{1}^{\prime }}{\left(Z_{A_{2}}\ldots Z_{A_{\xi +1}}|Z_{A_{1}}E^{\prime }\right)}_{\rho }-f_{1}\\ \geq H_{\text{min}}^{\epsilon_{1}}{\left(Z_{A_{1}}|Z_{A_{2}}Z_{A_{3}}\ldots Z_{A_{\xi +1}}E^{\prime }\right)}_{\rho }-f_{1}\\ +H_{\text{min}}^{\epsilon_{2}}{\left(Z_{A_{2}}|Z_{A_{1}}Z_{A_{3}}Z_{A4}\ldots Z_{A_{\xi +1}}E^{\prime }\right)}_{\rho }-f_{2}\\ +H_{\text{min}}^{\epsilon_{2}^{\prime }}{\left(Z_{A_{3}}Z_{A4}\ldots Z_{A_{\xi +1}}|Z_{A_{1}}Z_{A_{2}}E^{\prime }\right)}_{\rho }\\ \geq \ldots \\ \geq \sum_{i=1}^{\xi +1}\left[H_{\text{min}}^{\epsilon_{i}}{\left(Z_{A_{i}}|\left({∁}_{Z_{A}}Z_{A_{i}}\right)E^{\prime }\right)}_{\rho }\right]-\sum_{i=1}^{\xi }\left(f_{i}\right) \end{array}$$ (17)

where $f_{1}=2{\text{log}}_{2}\frac{1}{\epsilon -2\epsilon_{1}-\epsilon_{1^{\prime }}}$, $f_{i}=2{\text{log}}_{2}\frac{1}{\epsilon_{i-1^{\prime }}-2\epsilon_{i}-\epsilon_{1^{\prime }}}$ if i∈[2,ξ], $\epsilon_{\xi +1}=\epsilon_{\xi^{\prime }}$. Because $\left({∁}_{Z_{A}}Z_{A_{i}}\right)\in \left({∁}_{D_{A}}D_{A_{i}}\right)$, using data processing inequality, we can calculate that

$$\begin{array}{c} H_{\text{min}}^{\epsilon_{i}}{\left(Z_{A_{i}}|\left({∁}_{Z_{A}}Z_{A_{i}}\right)E^{\prime }\right)}_{\rho }=H_{\text{min}}^{\epsilon_{i}}{\left(Z_{A_{i}}|\left({∁}_{Z_{A}}Z_{A_{i}}\right)E^{\prime }\right)}_{\rho_{i}^{\prime }}\\ \geq H_{\text{min}}^{\epsilon_{i}}{\left(Z_{A_{i}}|\left({∁}_{D_{A}}D_{A_{i}}\right)E^{\prime }\right)}_{\rho_{i}^{\prime }} \end{array}$$ (18)

where $\rho_{i}^{\prime }$ denotes the quantum state in the protocol that include the space $Z_{A_{i}}$, $\left({∁}_{D_{A}}D_{A_{i}}\right)$, and $E^{\prime }$, and $\rho ={\text{Tr}}_{\left({∁}_{\left({∁}_{D_{A}}D_{A_{i}}\right)}\left({∁}_{Z_{A}}Z_{A_{i}}\right)\right)}\rho_{i}^{\prime }$. Thus, we can prove that, in the original protocol, there is

$$H_{\text{min}}^{\epsilon }{\left(Z_{A}|E^{\prime }\right)}_{\rho }\geq \sum_{i=1}^{\xi +1}\left[H_{\text{min}}^{\epsilon_{i}}{\left(Z_{A_{i}}|\left({∁}_{D_{A}}D_{A_{i}}\right)E^{\prime }\right)}_{\rho_{i}^{\prime }}\right]-\sum_{i=1}^{\xi }\left(f_{i}\right)$$ (19)

In addition, this leads us to Lemma 1.

Combined with the fact that $H_{\text{min}}^{{\tilde{\epsilon }}_{i}}{\left(Z_{A_{i^{\prime }}}|D_{A_{i^{\prime }}}E^{\prime }\right)}_{\rho_{i}^{\prime \prime }}\geq H_{\text{min}}^{{\tilde{\epsilon }}_{i}}{\left(Z_{A_{i^{\prime }}}|E^{\prime }\right)}_{\rho^{\prime }}$, we can calculate

$$H_{\text{min}}^{\epsilon }{\left(Z_{A}|E^{\prime }\right)}_{\rho }\geq \sum_{i=1}^{\xi +1}\left[H_{\text{min}}^{\epsilon_{i}}{\left(Z_{A_{i^{\prime }}}|E^{\prime }\right)}_{\rho^{\prime }}\right]-\sum_{i=1}^{\xi }\left(f_{i}\right)$$ (20)

where $f_{1}=2{\text{log}}_{2}\frac{1}{\epsilon -2\epsilon_{1}-\epsilon_{1^{\prime }}}$, $f_{i}=2{\text{log}}_{2}\frac{1}{\epsilon_{i-1^{\prime }}-2\epsilon_{i}-\epsilon_{i^{\prime }}}$ if i∈[2,ξ], $\epsilon_{\xi +1}=$ $\epsilon_{\xi^{\prime }}$, and this leads us to Lemma 2.

### Proof of Proposition 1

As we have discussed in the “Security analysis framework addressing correlated sources” section in Results, for most protocols, the smooth min-entropy can be estimated only for specific events in Z. In this case, by applying the chain-rule result (*43*) once again, we can obtain

$$\begin{array}{c} H_{\text{min}}^{{\tilde{\epsilon }}_{i}}{\left(Z_{A_{i^{\prime }}}|E^{\prime }\right)}_{\rho^{\prime }}\\ \geq H_{\text{min}}^{{\tilde{\epsilon }}_{i^{\prime }}}{\left(Z_{Z,A_{i^{\prime }}}|E^{\prime }\right)}_{\rho^{\prime }}+H_{\text{min}}^{{\tilde{\epsilon }}_{i}^{\prime \prime }}{\left(Z_{X,A_{i^{\prime }}}|Z_{Z,A_{i^{\prime }}}E^{\prime }\right)}_{\rho^{\prime }}+f_{i^{\prime }}\\ \geq H_{\text{min}}^{{\tilde{\epsilon }}_{i^{\prime }}}{\left(Z_{Z,A_{i^{\prime }}}|E^{\prime }\right)}_{\rho^{\prime }}+f_{i^{\prime }} \end{array}$$ (21)

where $Z_{A_{i^{\prime }}}=Z_{Z,A_{i^{\prime }}}Z_{X,A_{i^{\prime }}}$, in which $Z_{Z,A_{i^{\prime }}}$ denotes the parts that can estimated and $Z_{X,A_{i^{\prime }}}$ denotes the parts that cannot; by setting ${\tilde{\epsilon }}_{i^{\prime \prime }}=0$, $f_{i^{\prime }}$ then satisfies $f_{i^{\prime }}=2{\text{log}}_{2}\frac{1}{{\tilde{\epsilon }}_{i}-{\tilde{\epsilon }}_{i^{\prime }}}$. From the uncertainty relation and the parameter estimation of the QKD process (*51*), it is known that

$$H_{\text{min}}^{{\tilde{\epsilon }}_{i^{\prime }}}{\left(Z_{Z,A_{i^{\prime }}}|E^{\prime }\right)}_{\rho^{\prime }}\geq n_{i}\left(1-h\left({\bar{e_{i}}}_{{\tilde{\epsilon }}_{i^{\prime }}}^{U}\right)\right)$$ (22)

where $h\left(x\right)=-x{\text{log}}_{2}\left(x\right)-\left(1-x\right){\text{log}}_{2}\left(1-x\right)$, $n_{i}$ is the number of bits in $Z_{Z,A_{i^{\prime }}}$, and ${\bar{e_{i}}}_{\epsilon_{i}}^{U}$ is the upper bound of the phase error rate $e_{i}$ with the probability $p_{\text{fail}}^{i}$ of phase errors exceeding this bound that satisfies $p_{\text{fail}}^{i}={\left({\tilde{\epsilon }}_{i^{\prime }}\right)}^{2}$. For $Z_{A_{i^{\prime }}}$, let it contain $n=\sum_{i=1}^{\xi +1}n_{i}$ bits, and let the phase error rate be e, satisfying $e=\left(\sum_{i=1}^{\xi +1}n_{i}e_{i}\right)/\left(\sum_{i=1}^{\xi +1}n_{i}\right)$. Also, let $p_{\text{fail}}$ be the probability of phase error rate e exceeding the bound $\left(\sum_{i=1}^{\xi +1}n_{i}{\bar{e_{i}}}_{{\tilde{\epsilon }}_{i}^{\prime }}^{U}\right)/\left(\sum_{i=1}^{\xi +1}n_{i}\right)$. Through discussions related to probability theory, we find that, if each $e_{i}$ exceeds its upper bound, then e must exceed its upper bound; conversely, if none of the $e_{i}$’s exceed their upper bound, then e must also not exceed its upper bound. Therefore, we can derive the relationship between these probabilities as $\prod p_{\text{fail}}^{i}\leq p_{\text{fail}}\leq 1-\prod \left(1-p_{\text{fail}}^{i}\right)$. Therefore, because ${\bar{e}}_{\epsilon }^{U}$ is monotonically decreasing with respect to ϵ, we can conclude that

$${\bar{e}}_{\prod_{i=1}^{\xi +1}{\tilde{\epsilon }}_{i^{\prime }}}^{U}\geq \frac{\sum_{i=1}^{\xi +1}n_{i}{\bar{e_{i}}}_{{\tilde{\epsilon }}_{i^{\prime }}}^{U}}{\sum_{i=1}^{\xi +1}n_{i}}$$ (23)

Thus, combined with the concavity of the h(x) function, we can obtain that

$$\begin{array}{c} \sum_{i=1}^{\xi +1}H_{\text{min}}^{{\tilde{\epsilon }}_{i^{\prime }}}{\left(Z_{Z,A_{i^{\prime }}}|E^{\prime }\right)}_{\rho^{\prime }}\geq \left(\sum_{i=1}^{\xi +1}n_{i}\right)\left(1-h\left(\frac{\sum_{i=1}^{\xi +1}n_{i}{\bar{e_{i}}}_{{\tilde{\epsilon }}_{i^{\prime }}}^{U}}{\sum_{i=1}^{\xi +1}n_{i}}\right)\right)\\ \geq n\left(1-h\left({\bar{e}}_{\epsilon^{\prime }}^{U}\right)\right) \end{array}$$ (24)

where $\epsilon^{\prime }=\prod_{i=1}^{\xi +1}{\tilde{\epsilon }}_{i^{\prime }}$.

Combining Lemma 2 and Eqs. 21 and 24 and without loss of generality taking ${\tilde{\epsilon }}_{i}=\epsilon_{i}$, $f_{i}=f_{j}$, ${\tilde{\epsilon }}_{i^{\prime }}={\tilde{\epsilon }}_{j^{\prime }}$, and $\epsilon_{i}=\epsilon_{j}$, we can conclude that the minimum smooth entropy of the original protocol and the upper bound of the phase error estimated in the new protocol satisfy

$$H_{\text{min}}^{\epsilon }{\left(Z_{A}|E^{\prime }\right)}_{\rho }\geq n\left(1-h\left({\bar{e}}_{\hat{\epsilon }}^{U}\right)\right)-\xi f-\left(\xi +1\right)f^{\prime }$$ (25)

where ϵ, $\hat{\epsilon }$, and f satisfy $\hat{\epsilon }={\left(\frac{\epsilon -\xi \frac{1}{2^{f/2}}}{2\xi +1}-\frac{1}{2^{f^{\prime }/2}}\right)}^{\xi +1}$, and this leads us to Proposition 1.

### Proof that $|\Phi \rangle_{A}^{\text{equ}}$ ensures the security of $|\Phi \rangle_{A}$

The key of the secure proof of the secure QKD against correlated leakage source is to

prove that $|\Phi \rangle_{A}^{\text{equ}}$ ensures the security of $|\Phi \rangle_{A}$. We begin by considering the simplest case, where the correlation range ξ=1, and the total number of rounds N is even. The more general case is discussed in the Supplementary Text.

First, we will remove the correlation with the conclusion mentioned in Proposition 1. Treat the protocol in Eq. 7 as the original protocol described in Proposition 1, and, then, the new protocol without correlation can be expressed as

$$|\Phi \rangle_{A}^{\text{new}}=|\Phi \rangle_{A^{1}}⊗|\Phi \rangle_{A^{2}}$$ (26)

where $|{\Phi \rangle }_{A^{1}}=\left[\sum_{r_{1}^{\frac{N}{2}}{r^{\prime }}_{1}^{\frac{N}{2}}a_{1}^{N}}\left(\prod_{i=1}^{\frac{N}{2}}\sqrt{p_{r_{i}}p_{r_{i^{\prime }}}}\prod_{j=1}^{N}\sqrt{q_{a_{j}}}\right)({⊗}_{i=1}^{\frac{N}{2}}|r_{i^{\prime }}\rangle_{{A_{i}}^{\prime }}{⊗}_{j=1}^{N}|a_{i}\rangle_{A_{{i^{\prime }}^{\prime }}})\right]$ $\left({⊗}_{i=1}^{\frac{N}{2}}|r_{i}\rangle_{A_{i}}|\psi_{r_{i},r^{\prime }\left(i,1\right),a\left(i,1\right)}^{{\text{imp}}^{\prime }}\rangle_{C_{i^{\prime }}}\right)$ denotes the entanglement-equivalent protocol for the first time of N QKD rounds and ${|\Phi \rangle }_{A^{2}}=\left[\sum_{r_{\frac{N}{2}+1}^{N}{r^{\prime }}_{\frac{N}{2}+1}^{N}a_{n+1}^{2N}}\right.\left(\prod_{i=\frac{N}{2}+1}^{N}\sqrt{p_{r_{i}}p_{r_{i^{\prime }}}}\prod_{j=N+1}^{2N}\sqrt{q_{a_{j}}}\right)\left({⊗}_{\frac{N}{2}+1}^{N}{|r_{i^{\prime }}\rangle }_{A_{i}\prime }{⊗}_{j=N+1}^{2N}{|a_{i}\rangle }_{A_{{i^{\prime }}^{\prime }}}\right)$ $\left.\left({⊗}_{\frac{N}{2}+1}^{N}{|r_{i}\rangle }_{A_{i}}|\psi_{r_{i},r^{\prime }\left(i,1\right),a\left(i,1\right)}^{{\text{imp}}^{\prime }}\rangle_{C_{i^{\prime }}}\right)\right]$ denotes the entanglement-equivalent protocol for the next time of N QKD rounds, where $r_{i}$ denotes the encoding ancilla in *i*th key generation rounds, $r_{i}^{\prime }$ denotes the encoding ancilla in *i*th leakage rounds, $a_{i}$ denotes the purified ancilla in the *i*th physical rounds, and $|{\psi_{r_{i},r^{\prime }\left(i,\xi \right),a\left(i,\xi \right)}^{{\text{imp}}^{\prime }}\rangle }_{C_{i^{\prime }}}$ denotes the state sent into the channel in the *i*th key generation round and the following ξ physical rounds, where, in case $|{\Phi \rangle }_{A^{1}}$, $r^{\prime }\left(i,1\right)={r^{\prime }}_{i-1}^{i}$, $a\left(i,1\right)=a_{2i-2}^{2i}$, and, in case $|{\Phi \rangle }_{A^{2}}$, $r^{\prime }\left(i,1\right)={r^{\prime }}_{i}^{i+1}$, $a\left(i,1\right)=a_{2i-1}^{2i+1}$, satisfies

$${|\psi_{r_{i},r^{\prime }\left(i,1\right),a\left(i,1\right)}^{{\text{imp}}^{\prime }}\rangle }_{C_{i^{\prime }}}=\left\{\begin{array}{c} {|\psi_{r_{i}r_{i-1}^{\prime },a_{2i-2}^{2i-1}}^{\text{imp}}\rangle }_{C_{2i-1}}{|\psi_{r_{i}^{\prime }r_{i},a_{2i-1}^{2i}}^{\text{imp}}\rangle }_{C_{2i}},\text{in}\;\text{case}\;{|\Phi \rangle }_{A^{1}}\\ {|\psi_{r_{i}r_{i}^{\prime },a_{2i-1}^{2i}}^{\text{imp}}\rangle }_{C_{2i}}{|\psi_{r_{i+1}^{\prime }r_{i},a_{2i}^{2i+1}}^{\text{imp}}\rangle }_{C_{2i+1}},\text{in}\;\text{case}\;{|\Phi \rangle }_{A^{2}} \end{array}\right.$$ (27)

and where ${|\psi_{r_{i-\xi }^{i},a_{i-\xi }^{i}}^{\text{imp}}\rangle }_{C_{i}}$ is denoted in Eq. 7.

As discussed in Proposition 1, the new protocol can reveal all $r_{i}^{\prime }$ and *a_j_* to Eve. To further ensure that the new protocol is not only independently distributed but also i.i.d., we additionally require Alice to send an auxiliary quantum state ${|\psi_{r_{i},r^{\prime }\left(i,1\right),a\left(i,1\right)}^{\text{add}}\rangle }_{c_{i}^{\prime \prime }}$ in the $c_{i}^{\prime \prime }$ space for each *r_i_.* We denote this modified protocol as ${|\Phi \rangle }_{A}^{{\text{new}}_{2}}$. For any ${|\psi_{r_{i},r^{\prime }\left(i,1\right),a\left(i,1\right)}^{\text{add}}\rangle }_{c_{i}^{\prime \prime }}$, protocol ${|\Phi \rangle }_{A}^{{\text{new}}_{2}}$ guarantees the security of ${|\Phi \rangle }_{A}^{\text{new}}$ in Eq. 26. Thus, combined with Eq. 26, ${|\Phi \rangle }_{A}^{{\text{new}}_{2}}$ satisfies

$$\begin{array}{c} {|\Phi \rangle }_{A}^{{\text{new}}_{2}}=\left[\sum_{r_{1}^{\prime N}}\sum_{a_{1}^{2N}}\left(\prod_{i-1}^{N}\sqrt{p_{r_{i}^{\prime }}}\prod_{j=1}^{2N}\sqrt{q_{a_{j}}}\right)\left(\overset{N}{\underset{i=1}{⊗}}{|r_{i}^{\prime }\rangle }_{A_{i}^{\prime }}\overset{2N}{\underset{j=1}{⊗}}{|a_{j}\rangle }_{A_{i}^{\prime \prime }}\right)\right.\\ ⊗\left[\sum_{r_{1}^{N}}\left(\prod_{i=1}^{N}\sqrt{p_{r_{i}}}\right)\left(\overset{N}{\underset{i=1}{⊗}}{|r_{i}\rangle }_{A_{i}}{|\psi_{r_{i},r^{\prime }\left(i,1\right),a\left(i,1\right)}^{{\text{imp}}^{\prime }}\rangle }_{C_{i}^{\prime }}{|\psi_{r_{i},r^{\prime }\left(i,1\right),a\left(i,1\right)}^{\text{add}}\rangle }_{C_{i}^{\prime \prime }}\right)\right] \end{array}$$ (28)

After adding additional states ${|\psi_{r_{i},r^{\prime }\left(i,1\right),a\left(i,1\right)}^{\text{add}}\rangle }_{c_{i}^{\prime \prime }}$, our goal is to prove that there exist a choice of those states that can make the protocol i.i.d. after acting an unitary mapping on ${|\Phi \rangle }_{A}^{{\text{new}}_{2}}$. To achieve this, first, we should isolate the terms related to the *i*th coding ancilla *r_i_* in Eq. 28, denoted as ${|\Phi \rangle }_{A,\text{iso}\;r_{i}}^{{\text{new}}_{2,i}}$, which satisfies

$$\begin{array}{c} {|\Phi \rangle }_{A,\text{iso}r_{i}}^{{\text{new}}_{2,i}}=\\ \left\{ \begin{array}{c} \sum_{r^{\prime }\left(i,\xi \right)}\sum_{a\left(i,\xi \right)}\left(\prod_{j=i-1}^{i}\sqrt{p_{r_{j}^{\prime }}}\prod_{k=2i-2}^{2i}\sqrt{p_{a_{k}}}\right)\;\left(\overset{i}{\underset{j=i-1}{⊗}}{|r_{j}^{\prime }\rangle }_{A_{j}^{\prime }}\overset{i}{\underset{k=2i-2}{⊗}}{|a_{k}\rangle }_{A_{k}^{\prime \prime }}\right)\\ ⊗{|\psi_{r_{i},r^{\prime }\left(i,\xi \right),a\left(i,\xi \right)}^{\text{im}p^{\prime }}\rangle }_{C_{i}^{\prime }}{|\psi_{r_{i},r^{\prime }\left(i,\xi \right),a\left(i,\xi \right)}^{\text{add}}\rangle }_{C_{i}^{\prime \prime }},i\leq \frac{N}{2},\\ \sum_{r^{\prime }\left(i,\xi \right)}\sum_{a\left(i,\xi \right)}\left(\prod_{j=i}^{i+1}\sqrt{p_{r_{j}^{\prime }}}\prod_{k=2i-1}^{2i+1}\sqrt{p_{a_{k}}}\right)\left(\overset{i+1}{\underset{j=i}{⊗}}{|r_{j}^{\prime }\rangle }_{A_{j}^{\prime }}\overset{2i+1}{\underset{k=2i-1}{⊗}}{|a_{k}\rangle }_{A_{k}^{\prime \prime }}\right)\\ ⊗{|\psi_{r_{i},r^{\prime }\left(i,\xi \right),a\left(i,\xi \right)}^{{\text{imp}}^{\prime }}\rangle }_{C_{i}^{\prime }}{|\psi_{r_{i},r^{\prime }\left(i,\xi \right),a\left(i,\xi \right)}^{\text{add}}\rangle }_{C_{i}^{\prime \prime }},i\rangle \frac{N}{2} \end{array}\right. \end{array}$$ (29)

In the following discussion, we will take the case of *i* ≤ *N*/2, namely, the case ${|\Phi \rangle }_{A^{1}}$ as an example. The analysis for the other case proceeds analogously. Because ${|\psi_{r_{i},r^{\prime }\left(i,1\right),a\left(i,1\right)}^{\text{add}}\rangle }_{C_{i}^{\prime \prime }}$ is arbitrary, we can transfer part of its phase to ${|\psi_{r_{i},r^{\prime }\left(i,1\right),a\left(i,1\right)}^{{\text{imp}}^{\prime }}\rangle }_{C_{i}^{\prime }}$ to form ${|\psi_{r_{i},r^{\prime }\left(i,1\right),a\left(i,1\right)}^{{\text{imp}}^{\prime \prime }}\rangle }_{C_{i}^{\prime }}$ and ${|\psi_{r_{i},r^{\prime }\left(i,1\right),a\left(i,1\right)}^{{\text{add}}^{\prime }}\rangle }_{C_{i}^{\prime \prime }}$, such that $\langle 0{|\psi_{r_{i},r^{\prime }\left(i,1\right),a\left(i,1\right)}^{{\text{imp}}^{\prime \prime }}\rangle }_{C_{i}^{\prime }}$ is real and positive, where ${|0\rangle }_{C_{i}^{\prime }}={|0\rangle }_{C_{2i-1}}{|0\rangle }_{C_{2i}}$ and ${|0\rangle }_{C_{j}}$ is the vacuum state in the 𝑗th physical rounds. For further analysis, we additionally define two states, respectively, satisfying

$$\begin{array}{c} {|\Phi \rangle }_{A,\text{iso}r_{i}}^{\text{new},i}=\\ \left[\sum_{r^{\prime }\left(i,\xi \right)}\sum_{a\left(i,\xi \right)}\left(\prod_{j=i-1}^{i}\sqrt{p_{r_{j}^{\prime }}}\prod_{k=2i-2}^{2i}\sqrt{p_{a_{k}}}\right)\left(\overset{i}{\underset{j=i-1}{⊗}}{|r_{j}^{\prime }\rangle }_{A_{j}^{\prime }}\overset{2i}{\underset{k=2i-2}{⊗}}{|a_{k}\rangle }_{A_{k}^{\prime \prime }}\right){|\psi_{r_{i},r\prime \left(i,\xi \right),a\left(i,\xi \right)}^{{\text{imp}}^{\prime \prime }}\rangle }_{C_{i}^{\prime }}\right]\\ {|\Phi \rangle }_{A,\text{vac}}^{\text{new},i}=\left[\sum_{r^{\prime }\left(i,\xi \right)}\sum_{a\left(i,\xi \right)}\left(\prod_{j=i-1}^{i}\sqrt{p_{r_{j}^{\prime }}}\prod_{k=2i-2}^{2i}\sqrt{p_{a_{k}}}\right)\left(\overset{i}{\underset{j=i-1}{⊗}}{|r_{j}^{\prime }\rangle }_{A_{j}^{\prime }}\overset{2i}{\underset{k=2i-2}{⊗}}{|a_{k}\rangle }_{A_{k}^{\prime \prime }}\right){|0\rangle }_{C_{i}^{\prime }}\right] \end{array}$$ (30)

Thus, due to Assumption 2, which further leads to $\underset{r_{i-\xi }^{i-1}}{\text{min}}\underset{a_{i-\xi }^{i}}{\text{min}}\left(\langle 0|\psi_{r_{i-\xi }^{i},a_{i-\xi }^{i}}^{\text{imp}}\rangle {\langle \psi_{r_{i-\xi }^{i},a_{i-\xi }^{i}}^{\text{imp}}|}_{C_{i}}|0\rangle \right)\geq V_{r_{i}}^{A}$, the two intermediate states defined in Eq. 30 satisfy

$$\begin{array}{c} {\langle \Phi |}_{A,\text{iso}r_{i}}^{\text{new},i}{|\Phi \rangle }_{A,\text{vac}}^{\text{new},i}=|\sum_{r^{\prime }\left(i,\xi \right)}\sum_{a\left(i,\xi \right)}\left(\prod_{j=i-1}^{i}p_{r_{j}^{\prime }}\prod_{k=2i-2}^{2i}p_{a_{k}}\right)\langle \psi_{r_{i},r^{\prime }\left(i,\xi \right),a\left(i,\xi \right)}^{{\text{imp}}^{\prime \prime }}|{0\rangle }_{C_{i}^{\prime }}|\\ \geq \sum_{r^{\prime }\left(i,\xi \right)}\sum_{a\left(i,\xi \right)}\left(\prod_{j=i-1}^{i}p_{r_{j}^{\prime }}\prod_{k=2i-2}^{2i}p_{a_{k}}\right)\left(\sqrt{V_{r_{i}}^{A}}\prod_{j=i-1}^{i}\sqrt{V_{r_{i}^{\prime }}^{A}}\right)\\ =\sqrt{V_{r_{i}}^{A}}\left(p_{0}\sqrt{V_{0}^{A}}+p_{1}\sqrt{V_{1}^{A}}\right)≕\sqrt{V_{r_{i}}^{A,1}} \end{array}$$ (31)

Because Eq. 31 satisfies for both 𝑟_𝑖_ = 0 and 1, we can find that

$$|{\langle \Phi |}_{A,\text{iso}\;0}^{\text{new},i}{|\Phi \rangle }_{A,\text{iso}\;1}^{\text{new},i}|\geq \sqrt{V_{0}^{A,1}V_{1}^{A,1}}-\sqrt{\left(1-V_{0}^{A,1}\right)\left(1-V_{1}^{A,1}\right)}$$ (32)

In addition, from Eqs. 29 and 31, we have that

$$\begin{array}{c} {\langle \Phi |}_{A,\mathrm{iso}0}^{\mathrm{ne}w_{2},i}{|\Phi \rangle }_{A,\mathrm{iso}1}^{\mathrm{ne}w_{2},i}\\ =\sum_{r^{\prime }\left(i,\xi \right)}\sum_{a\left(i,\xi \right)}\left(\prod_{j=i-1}^{i}p_{r_{j}^{\prime }}\prod_{k=2i-2}^{2i}p_{a_{k}}\right)\langle \psi_{0,r^{\prime }\left(i,\xi \right),a\left(i,\xi \right)}^{{\text{imp}}^{\prime \prime }}|{\psi_{1,r^{\prime }\left(i,\xi \right),a\left(i,\xi \right)}^{{\text{imp}}^{\prime \prime }}\rangle }_{C_{i}^{\prime }}\langle \psi_{0,r^{\prime }\left(i,\xi \right),a\left(i,\xi \right)}^{{\text{add}}^{\prime }}|{\psi_{1,r^{\prime }\left(i,\xi \right),a\left(i,\xi \right)}^{{\text{add}}^{\prime }}\rangle }_{C_{i}^{\prime \prime }}\\ {\langle \Phi |}_{A,\mathrm{iso}0}^{\mathrm{new},i}{|\Phi \rangle }_{A,\mathrm{iso}1}^{\mathrm{new},i}=\\ \sum_{r^{\prime }\left(i,\xi \right)}\sum_{a\left(i,\xi \right)}\left(\prod_{j=i-1}^{i}p_{r_{j}^{\prime }}\prod_{k=2i-2}^{2i}p_{a_{k}}\right)\langle \psi_{0,r^{\prime }\left(i,\xi \right),a\left(i,\xi \right)}^{{\text{imp}}^{\prime \prime }}|{\psi_{1,r^{\prime }\left(i,\xi \right),a\left(i,\xi \right)}^{{\text{imp}}^{\prime \prime }}\rangle }_{C_{i}^{\prime }} \end{array}$$ (33)

Because $|\langle \psi_{0,r^{\prime }\left(i,\xi \right),a\left(i,\xi \right)}^{{\text{add}}^{\prime }}|{\psi_{1,r^{\prime }\left(i,\xi \right),a\left(i,\xi \right)}^{{\text{add}}^{\prime }}\rangle }_{C_{i}^{\prime \prime }}|\leq 1$, combined with Eq. 32, we can select a specific set of ${|\psi_{r_{i},r^{\prime }\left(i,\xi \right),a\left(i,\xi \right)}^{{\text{add}}^{\prime }}\rangle }_{C_{i}^{\prime \prime }}$ such that

$${\langle \Phi |}_{A,\text{iso}\;0}^{{\text{new}}_{2},i}{|\Phi \rangle }_{A,\text{iso}\;1}^{{\text{new}}_{2},i}=\sqrt{V_{0}^{A,1}V_{1}^{A,1}}-\sqrt{\left(1-V_{0}^{A,1}\right)\left(1-V_{1}^{A,1}\right)}$$ (34)

and same proof satisfies when satisfies when 𝑖 > 𝑁/2. Then, we will prove that this set of ${|\psi_{r_{i},r^{\prime }\left(i,\xi \right),a\left(i,\xi \right)}^{{\text{add}}^{\prime }}\rangle }_{C_{i}^{\prime \prime }}$ can make the protocol i.i.d.

Specifically, we construct this i.i.d. protocol as equivalent protocol ${|\Phi \rangle }_{A}^{\text{equ}}$, which satisfies

$$\begin{array}{c} {|\Phi \rangle }_{A}^{\text{equ}}=\left(\prod_{i=1}^{N}U_{j}\right){|\Phi \rangle }_{A}^{{\text{new}}_{2}}\\ =\left[\sum_{{r^{\prime }}_{1}^{\xi N}}\sum_{a_{1}^{\left(1+\xi \right)N}}\left(\prod_{i=1}^{N}\sqrt{p_{r_{i}^{\prime }}}\prod_{j=1}^{2N}\sqrt{q_{a_{j}}}\right)\left(\overset{N}{\underset{i=1}{⊗}}{|r_{i}^{\prime }\rangle }_{A_{i}^{\prime }}\overset{2N}{\underset{j=1}{⊗}}{|a_{i}\rangle }_{A_{i}^{\prime \prime }}\right)\right.\\ \left.⊗\sum_{r_{1}^{N}}\left(\prod_{i=1}^{N}\sqrt{p_{r_{i}^{\prime }}}\right)\left(\overset{N}{\underset{i=1}{⊗}}{|r_{i}\rangle }_{A_{i}}{|\psi_{r_{i}}^{\text{equ}}\rangle }_{C_{i}^{\prime \prime \prime }}\right)\right] \end{array}$$ (35)

where **U**_𝑖_ is an unitary mapping from the space $\cup {}_{j=i-1}^{i}A_{j}^{\prime }\cup {}_{2i-2}^{2i}{A^{\prime }}_{k}^{\prime \prime }\cup C_{i}^{\prime }\cup C_{i}^{\prime \prime }$ into $\cup {}_{j=i-1}^{i}A_{j}^{\prime }\cup {}_{2i-2}^{2i}A_{k}^{\prime \prime }\cup C_{i}^{\prime \prime \prime }$ when 𝑖 ≤ 𝑁/2 and unitary mapping from the space $\cup {}_{j=i}^{i+1}A_{j}^{\prime }\cup {}_{2i-2}^{2i+1}A_{k}^{\prime \prime }\cup C_{i}^{\prime }C_{i}^{\prime \prime }$ into $\cup {}_{j=i}^{i+1}A_{j}^{\prime }\cup {}_{2i-1}^{2i+1}A_{k}^{\prime \prime }\cup C_{i}^{\prime \prime \prime }$ when 𝑖 ≤ 𝑁/2, which satisfies

$$\begin{array}{c} U_{i}{|\Phi \rangle }_{A,\text{iso}r_{i}}^{{\text{new}}_{2},i}=\\ \left\{\begin{array}{c} \sum_{r^{\prime }\left(i,\xi \right)}\sum_{a\left(i,\xi \right)}\left(\prod_{j=i-1}^{i}\sqrt{p_{r_{i}^{\prime }}}\prod_{k=2i-2}^{2i}\sqrt{p_{a_{k}}}\right)\left(\overset{i}{\underset{j=i-1}{⊗}}{|r_{i}^{\prime }\rangle }_{A_{j}^{\prime }}\overset{2i}{\underset{k=2i-2}{⊗}}{|a_{k}\rangle }_{A_{k}^{\prime \prime }}\right){|\psi_{r_{i}}^{\text{equ}}\rangle }_{C_{i}^{\prime \prime \prime }},i\leq \frac{N}{2}\\ \sum_{r^{\prime }\left(i,\xi \right)}\sum_{a\left(i,\xi \right)}\left(\prod_{j=i}^{i+1}\sqrt{p_{r_{i}^{\prime }}}\prod_{k=2i-1}^{2i+1}\sqrt{p_{a_{k}}}\right)\left(\overset{i+1}{\underset{j=i}{⊗}}{|r_{i}^{\prime }\rangle }_{A_{j}^{\prime }}\overset{2i+1}{\underset{k=2i-1}{⊗}}{|a_{k}\rangle }_{A_{k}^{\prime \prime }}\right){|\psi_{r_{i}}^{\text{equ}}\rangle }_{C_{i}^{\prime \prime \prime }},i\rangle \frac{N}{2} \end{array}\right. \end{array}$$ (36)

In both two cases, ${|\psi_{r_{i}}^{\text{equ}}\rangle }_{C_{i}^{\prime \prime \prime }}$ in Eq. 36 has no relation with $r_{j}^{\prime }$ and *a_k_*, so it is clear that 〈Φ∣A,iso 0new2,iUiUi†∣Φ〉A,iso 1new2,i=〈ψ0equ∣ψ1equ〉Ci‴. Thus, **U***_i_* exists if and only if ${\langle \Phi |{}_{A,\text{iso}\;0}^{{\text{new}}_{2},i}|\Phi \rangle }_{A,\text{iso}\;1}^{{\text{new}}_{2},i}=\langle \psi_{0}^{\text{equ}}{|\psi_{1}^{\text{equ}}\rangle }_{C_{i}^{\prime \prime \prime }}$. Without loss of generality, we assume that ${|\psi_{0}^{\text{equ}}\rangle }_{C_{i}^{\prime \prime \prime }}=|0\rangle$, ${|\psi_{1}^{\text{equ}}\rangle }_{C_{i}^{\prime \prime \prime }}=|\mu_{\text{equ}}\rangle$, where ∣0〉 is the vacuum state and $|\mu_{\text{equ}}\rangle$ is the coherent state with an average number of photons equals to μ_equ_. Combined with Eq. 34, we can calculate that μ_equ_ satisfies

$$e^{-\text{μequ}}={\left[\sqrt{V_{0}^{A,1}V_{1}^{A,1}}-\sqrt{\left(1-V_{0}^{A,1}\right)\left(1-V_{1}^{A,1}\right)}\right]}^{2}$$ (37)

Furthermore, observing that, in protocol ${|\Phi \rangle }_{A}^{\text{equ}}$, *r*′ and *a* no longer play any role, we simplify the equivalent protocol ${|\Phi \rangle }_{A}^{\text{equ}}$ by removing them. The final equivalent protocol then satisfies

$${|\Phi \rangle }_{A}^{\text{equ}}=\left[\sum_{r_{1}^{N}}\left(\prod_{i=1}^{N}\sqrt{p_{r_{i}}}\right)\left(\overset{N}{\underset{i=1}{⊗}}{|r_{i}\rangle }_{A_{i}}{|\psi_{r_{i}}^{\text{equ}}\rangle }_{C_{i}^{\prime \prime \prime }}\right)\right]$$ (38)

The above analysis similarly applies to more general values of ξ, and the detailed procedure can be found in the Supplementary Text. Ultimately, this leads to a conclusion of the form presented in Eq. 9.

### Phase error estimation of secure QKD against correlated leakage source

In the protocol described by Eq. 10, similar to the original protocol, a Z event occurs when exactly one of Alice or Bob chooses $r_{i}=0$, an O event occurs when both Alice and Bob choose $r_{i}=0$, and a B event occurs when both Alice and Bob choose $r_{i}=1$. In this case, we define the phase error for a Z event as ${|++\rangle }_{A_{i}B_{i}}=(\frac{{|0\rangle }_{A_{i}}+{|1\rangle }_{A_{i}}}{\sqrt{2}})⊗\left(\frac{{|0\rangle }_{B_{i}}+{|1\rangle }_{B_{i}}}{\sqrt{2}}\right)$ and ${|--\rangle }_{A_{i}B_{i}}=(\frac{{|0\rangle }_{A_{i}}-{|1\rangle }_{A_{i}}}{\sqrt{2}})⊗\left(\frac{{|0\rangle }_{B_{i}}-{|0\rangle }_{B_{i}}}{\sqrt{2}}\right)$. Specifically, a phase error is said to occur when Alice and Bob measure the state $\frac{{|++\rangle }_{A_{i}B_{i}}-{|--\rangle }_{A_{i}B_{i}}}{\sqrt{2}}=\frac{{|0\rangle }_{A_{i}}{|1\rangle }_{B_{i}}+{|1\rangle }_{A_{i}}{|0\rangle }_{B_{i}}}{\sqrt{2}}$ (*30*, *31*).

Because we do not assume Charlie to be trusted, we can further consider the case where Charlie is entirely controlled by Eve. In this scenario, Eve’s attack and Charlie’s measurement can be treated jointly. For simplicity, we refer to this combined operation as Eve’s positive operator-valued measurement (PVOM) $M_{c_{0}^{N}}^{E}$ in the following discussion, where $c_{0}^{N}=c_{0}c_{1}\ldots c_{N}$ denotes the measurement result announced by Charlie over total *n* rounds, where $c_{i}\in \left\{0,1\right\}$ represents the measurement result in the *i*th round, where 1 indicates a successful measurement and 0 indicates other measurement results, including successful measurement and conclusive results. First, consider a collective attack. In this scenario, Eve’s measurement can be represented as $M_{c_{0}^{N}}^{E}={⊗}_{i=1}^{N}M_{c_{i}}^{E,i}$, where $M_{c_{i}}^{E,i}$ is a Eve’s PVOM in the *i*th round, and $\forall i,j\in \left[0,N\right],\text{if}\;c_{i}=c_{j},M_{c_{i}}^{E,i}=M_{c_{j}}^{E,j}$. It is worth noting that Eve does not have access to the encoding ancilla spaces of Alice and Bob. Therefore, the phase error probability *P*_ph_ can be shown as

$$\begin{array}{c} P_{\text{ph}}=\text{Tr}\left(\left(P\left[\frac{{|0\rangle }_{A_{i}}{|1\rangle }_{B_{i}}+{|1\rangle }_{A_{i}}{|0\rangle }_{B_{i}}}{\sqrt{2}}\right]⊗M_{1}^{E,i}\right)⊗P\left[{|\Phi \rangle }_{i}^{\text{equ}}\right]⊗|0\rangle {\langle 0|}_{{\text{PE}}_{i}}\right)\\ =\frac{p_{0}p_{1}\left(1-p_{\text{PE}}\right)}{2}\text{Tr}\left(M_{1}^{E,i}P\left[{|\psi_{0}^{\text{Aequ}}\rangle }_{C_{i}^{A}}{|\psi_{1}^{\text{Bequ}}\rangle }_{C_{i}^{B}}+{|\psi_{1}^{\text{Aequ}}\rangle }_{C_{i}^{A}}{|\psi_{0}^{\text{Bequ}}\rangle }_{C_{i}^{B}}\right]\right) \end{array}$$ (39)

where P[∣⋅〉]=∣⋅〉〈⋅∣ and ${|\Phi \rangle }_{i}^{\text{equ}}=\left[\sum_{r_{i}\in \left\{0,1\right\}}\sqrt{p_{r_{i}}}{|r\rangle }_{iA_{i}}{|\psi_{r_{i}}^{\text{Aequ}}\rangle }_{C_{i}^{A}}\right]⊗\left[\sum_{r_{i}\in \left\{0,1\right\}}\sqrt{p_{r_{i}}}{|r_{i}\rangle }_{B_{i}}{|\psi_{r_{i}}^{\text{Bequ}}\rangle }_{C_{i}^{B}}\right]⊗\left[\sum_{m_{i}}\sqrt{p_{m_{i}}^{\text{PE}}}{|m_{i}\rangle }_{{\text{PE}}_{i}}\right]$ denotes the entanglement-equivalent protocol in the *i*th round, which satisfies $P\left[{|\Phi \rangle }^{\text{equ}}\right]={⊗}_{i=1}^{N}P\left[{|\Phi \rangle }_{i}^{\text{equ}}\right]$. Similarly, we can calculate the probabilities of the O event and the B event, denoted as $P_{O}$ and $P_{B}$, which satisfy

$$\begin{array}{c} P_{O}=\text{Tr}\left(\left(P\left[{|0\rangle }_{A_{i}}{|0\rangle }_{B_{i}}\right]⊗M_{1}^{E,i}\right)⊗P\left[{|\Phi \rangle }_{i}^{\text{equ}}\right]\right)\\ =\left(1-p_{\text{PE}}\right){\left(p_{0}\right)}^{2}\text{Tr}\left(M_{1}^{E,i}P\left[{|\psi_{0}^{\text{Aequ}}\rangle }_{C_{i}^{A}}{|\psi_{0}^{\text{Bequ}}\rangle }_{C_{i}^{B}}\right]\right)\\ P_{B}=\text{Tr}\left(\left(P\left[{|1\rangle }_{A_{i}}{|1\rangle }_{B_{i}}\right]⊗M_{1}^{E,i}\right)⊗P\left[{|\Phi \rangle }_{i}^{\text{equ}}\right]\right)\\ =\left(1-p_{\text{PE}}\right){\left(p_{1}\right)}^{2}\text{Tr}\left(M_{1}^{E,i}P\left[\psi {|\psi_{1}^{\text{Aequ}}\rangle }_{C_{i}^{A}}{|\psi_{1}^{\text{Bequ}}\rangle }_{C_{i}^{B}}\right]\right) \end{array}$$ (40)

As calculated in the existing works (*28*–*31*), the actually transmitted state satisfies

$$\begin{array}{c} {|\psi_{0}^{\text{Aequ}}\rangle }_{C_{i}^{A}}{|\psi_{1}^{\text{Bequ}}\rangle }_{C_{i}^{B}}+{|\psi_{1}^{\text{Aequ}}\rangle }_{C_{i}^{A}}{|\psi_{0}^{\text{Bequ}}\rangle }_{C_{i}^{B}}\\ =c_{0}{|\psi_{0}^{\text{Aequ}}\rangle }_{C_{i}^{A}}{|\psi_{0}^{\text{Bequ}}\rangle }_{C_{i}^{B}}+c_{1}{|\psi_{1}^{\text{Aequ}}\rangle }_{C_{i}^{A}}{|\psi_{1}^{\text{Bequ}}\rangle }_{C_{i}^{B}}+{\bar{c}}_{2}|\phi_{2}\rangle \end{array}$$ (41)

from Eq. 10, where $c_{0},c_{1}\rangle 0$, $c_{0}c_{1}=1$, ${\bar{c}}_{2}=\sqrt{\left(c_{0}+c_{1}-2e^{-\frac{\mu_{\text{equ}}^{A}}{2}}\right)\left(c_{0}+c_{1}-2e^{-\frac{\mu_{\text{equ}}^{B}}{2}}\right)}$, and $|\phi_{2}\rangle$ is a normalized state that keep Eq. 41 holds. Although the values of *c*_0_ and *c*_1_ can be optimized to improve the protocol’s performance, we assume $c_{0}=e^{-\left(\mu_{\text{equ}}^{A}+\mu_{\text{equ}}^{B}\right)/4}$ and $c_{1}=1/c_{0}$ to simplify the analysis. For $M_{1}^{E,i}$ to be positive, we can expand its eigenvalues and rewrite it as $M_{1}^{E,i}=\sum_{j}P\left[{|1\rangle }_{j}^{E,i}\right]$, where ${|1\rangle }_{j}^{E,i}$ is a nonnormalized eigenvector and $\text{Tr}\left(P\left[{|1\rangle }_{j}^{E,i}\right]\right)$ is the corresponding eigenvalue, which satisfies $\text{Tr}\sum_{j}\left(P\left[{|1\rangle }_{j}^{E,i}\right]\right)=\text{Tr}\left(M_{1}^{E,i}\right)\leq 1$. Then, Eq. 40 can be rewritten as

$$\begin{array}{c} P_{O}=\left(1-p_{\text{PE}}\right){\left(p_{0}\right)}^{2}\sum_{j}{|{\langle 1|}_{j}^{E,i}{|\psi_{0}^{\text{Aequ}}\rangle }_{C_{i}^{A}}{|\psi_{0}^{\text{Bequ}}\rangle }_{C_{i}^{B}}|}^{2}\\ P_{B}=\left(1-p_{\text{PE}}\right){\left(p_{1}\right)}^{2}\sum_{j}{|{\langle 1|}_{j}^{E,i}{|\psi_{1}^{\text{Aequ}}\rangle }_{C_{i}^{A}}{|\psi_{1}^{\text{Bequ}}\rangle }_{C_{i}^{B}}|}^{2} \end{array}$$ (42)

Combining Eqs. 39 and 41, we can calculate that

$$\begin{array}{c} \frac{2P_{\text{ph}}}{p_{0}p_{1}\left(1-p_{\text{PE}}\right)}=\\ \sum_{j}{|{\langle 1|}_{j}^{E,i}\left(c_{0}{|\psi_{0}^{\text{Aequ}}\rangle }_{C_{i}^{A}}{|\psi_{0}^{\text{Bequ}}\rangle }_{C_{i}^{B}}+c_{1}{|\psi_{1}^{\text{Aequ}}\rangle }_{C_{i}^{A}}{|\psi_{1}^{\text{Bequ}}\rangle }_{C_{i}^{B}}+{\bar{c}}_{2}|\phi_{2}\rangle \right)|}^{2}\\ \leq \sum_{j}\left(c_{0}^{2}{|{\langle 1|}_{j}^{E,i}{|\psi_{0}^{\text{Aequ}}\rangle }_{C_{i}^{A}}{|\psi_{0}^{\text{Bequ}}\rangle }_{C_{i}^{B}}|}^{2}+c_{1}^{2}{|{\langle 1|}_{j}^{E,i}{|\psi_{1}^{\text{Aequ}}\rangle }_{C_{i}^{A}}{|\psi_{1}^{\text{Bequ}}\rangle }_{C_{i}^{B}}|}^{2}+{\bar{c}}_{2}^{2}{|{\langle 1|}_{j}^{E,i}{\bar{c}}_{2}|\phi_{2}\rangle |}^{2}\right.\\ +2c_{0}c_{1}|{\langle 1|}_{j}^{E,i}{|\psi_{0}^{\text{Aequ}}\rangle }_{C_{i}^{A}}{|\psi_{0}^{\text{Bequ}}\rangle }_{C_{i}^{B}}||{\langle 1|}_{j}^{E,i}{|\psi_{1}^{\text{Aequ}}\rangle }_{C_{i}^{A}}{|\psi_{1}^{\text{Bequ}}\rangle }_{C_{i}^{B}}|\\ +2c_{0}{\bar{c}}_{2}|{\langle 1|}_{j}^{E,i}{|\psi_{0}^{\text{Aequ}}\rangle }_{C_{i}^{A}}{|\psi_{0}^{\text{Bequ}}\rangle }_{C_{i}^{B}}||{\langle 1|}_{j}^{E,i}{\bar{c}}_{2}|\phi_{2}\rangle |\\ \left.+2c_{1}{\bar{c}}_{2}|{\langle 1|}_{j}^{E,i}{|\psi_{1}^{\text{Aequ}}\rangle }_{C_{i}^{A}}{|\psi_{1}^{\text{Bequ}}\rangle }_{C_{i}^{B}}||{\langle 1|}_{j}^{E,i}{\bar{c}}_{2}|\phi_{2}\rangle |\right) \end{array}$$ (43)

From Cauchy-Schwarz inequality, we can see that ${\left(\sum_{i}x_{i}y_{i}\right)}^{2}\leq {\left(\sum_{i}x_{i}\right)}^{2}{\left(\sum_{i}y_{i}\right)}^{2}$; then, combined with the fact that $\sum_{j}{|{\langle 1|}_{j}^{E,i}{\bar{c}}_{2}|\phi_{2}\rangle |}^{2}\leq 1$, from Eqs. 42 and 43, we can calculate the upper bound of $P_{\text{ph}}$, which satisfies (*28*, *31*)

$$P_{\text{ph}}\leq \frac{p_{1}p_{0}}{2}\left(c_{0}^{2}\frac{P_{O}}{{\left(p_{0}\right)}^{2}}+c_{1}^{2}\frac{P_{B}}{{\left(p_{1}\right)}^{2}}+{\bar{c}}_{2}^{2}+2c_{0}c_{1}\sqrt{\frac{P_{O}P_{B}}{{\left(p_{0}\right)}^{2}{\left(p_{1}\right)}^{2}}}+c_{0}{\bar{c}}_{2}\sqrt{\frac{P_{O}}{{\left(p_{0}\right)}^{2}}}+c_{1}{\bar{c}}_{2}\sqrt{\frac{P_{B}}{{\left(p_{1}\right)}^{2}}}\right)$$ (44)

and this leads us to Eq. 11.
