# Supplementary material for: Secure quantum key distribution against correlated leakage source
Source: Sci Adv. 2026 Apr 10;12(15):eaed2420. doi: 10.1126/sciadv.aed2420 (PMC13068060; doi:10.1126/sciadv.aed2420)
Supplement: Supplementary file 1 — Supplementary Text [file sciadv.aed2420_sm.pdf]

Science Advances
AAAS

Supplementary Materials for

Secure quantum key distribution against correlated leakage source

Jia-Xuan Li *et al.*

Corresponding author: Zhen-Qiang Yin, yinzq@ustc.edu.cn; Shuang Wang, wshuang@ustc.edu.cn

Sci. Adv. **12**, eaed2420 (2026)
DOI: 10.1126/sciadv.aed2420

This PDF file includes:

Supplementary Text

Supplementary Text

In this supplementary text, we will prove that for more general values of ξ, there still exists a $|\Phi\rangle_{\mathrm{A}}^{\mathrm{equ}}$ that ensures the security of $|\Phi\rangle_{\mathrm{A}}$.

Recalling the main text, we first have Proposition 2 under Assumption 3, stated as follows.

Assumption 3. *The correlation is constrained within a maximum range ξ.*

Definition 2. *For any original protocol with a maximum correlation range ξ, we define a corresponding new protocol by repeating the original protocol $\xi + 1$ times. In the i-th repetition, only the rounds whose indices satisfy modulo $\xi + 1$ congruent to i (with the remainder $\xi + 1$ interpreted as 0) are used for key generation, while all other rounds are disclosed. The raw key of original protocol is denoted by $\mathbf{Z}_A$, the raw key of the i-th repetition of the new protocol is denoted by $\mathbf{Z}'_{A_i}$ and the the raw key of the whole new protocol is denoted by $\mathbf{Z}'_A$.*

Proposition 2. *The lower bond of the smooth min-entropy $H^{\epsilon}_{\min}(\mathbf{Z}_A|\mathbf{E}')_\rho$ of an original protocol with a maximum correlation range ξ can be bond by the upper bond of the estimation of phase error rate $\overline{e}^{\mathrm{U}}_{\widehat{\epsilon}}$ of the new protocol, where the definition of new protocol is in Definition 1. This relation satisfies*

$$H^{\epsilon}_{\min}(\mathbf{Z}_A|\mathbf{E}')_\rho \geq n\left(1 - h\left(\overline{e}^{\mathrm{U}}_{\widehat{\epsilon}}\right)\right) - \xi f - (\xi + 1)\, f', \tag{S1}$$

where ϵ, $\widehat{\epsilon}$ and f satisfy $\widehat{\epsilon} = \left(\frac{\epsilon - \xi \frac{1}{2^{f/2}}}{2\xi + 1} - \frac{1}{2^{f'/2}}\right)^{\xi+1}$.

Base on Proposition 2, we demonstrated in the main text the security of the two-state SNS protocol (*28–31*) under correlation, assuming that both Assumption 3 and Assumption 4 are satisfied. Assumption 4 is presented below.

Assumption 4. *For the two-state SNS-QKD protocol, the lower bound of the proportion of vacuum states in each round, under both the send and not send scenarios, is known. Specifically, given the i-th round and its preceding ξ rounds, the state sent into the channel during the current round $\rho^{\mathrm{A(B)}}_{\mathbf{r}^i_{i-\xi}, \mathbf{a}^i_{i-\xi}}$ satisfies*

$$\min_{\mathbf{r}^{i-1}_{i-\xi}} \left(\min_{\mathbf{a}^i_{i-\xi}} \left(\left| \langle 0 | \, \rho^{\mathrm{A(B)}}_{\mathbf{r}^i_{i-\xi}, \mathbf{a}^i_{i-\xi}} \, | 0 \rangle \right| \right) \right) \geq V^{\mathrm{A(B)}}_{r_i}, \tag{S2}$$

where $r_i \in \{0, 1\}$ denotes the encoding setting in i-th round, a_i denotes the set of ancillas in the system that are potentially related to rounds and can influence the transmitted state, which includes

controls over SPF, correlation, side-channel and so on, $V_{r_i}^{\mathrm{A(B)}}$ *denotes the lower bound of the proportion of vacuum states and the sequence from i-th to the j-th round for a and r are defined as* $a_i^j := a_j a_{j-1} \ldots a_i$ *and* $r_i^j := r_j r_{j-1} \ldots r_i$ *respectively.*

The main text has already provided the proof for the case where $\xi = 1$. Here, we present the proof for the general case where ξ takes arbitrary values. In this scenario, the entanglement-based equivalent protocol on Alice's side satisfies

$$|\Phi\rangle_{\mathrm{A}} = \left[\sum_{\mathbf{r}_1^N \mathbf{a}_1^N} \left(\prod_{i=1}^{N} \sqrt{p_{r_i} q_{a_i}}\right) \left(\bigotimes_{i=1}^{N} |r_i\rangle_{A_i} |a_i\rangle_{A_i''} \left|\psi^{\mathrm{imp}}_{\mathbf{r}_{i-\xi}^i, \mathbf{a}_{i-\xi}^i}\right\rangle_{C_i}\right)\right], \tag{S3}$$

where q_{a_i} is the the probability of selecting a_i. Recall the Eq. (S2) of Assumption 4, it can be rewritten as

$$\min_{\mathbf{r}_{i-\xi}^{i-1}} \min_{\mathbf{a}_{i-\xi}^i} \left(\left\langle 0 \middle| \psi^{\mathrm{imp}}_{\mathbf{r}_{i-\xi}^i, \mathbf{a}_{i-\xi}^i} \right\rangle \left\langle \psi^{\mathrm{imp}}_{\mathbf{r}_{i-\xi}^i, \mathbf{a}_{i-\xi}^i} \middle|_{C_i} |0\rangle\right) \geq V_{r_i}^{\mathrm{A}}. \tag{S4}$$

Treat the protocol in Eq. (S3) as the *original protocol* described in Proposition 2, then the *new protocol* without correlation can be expressed as

$$|\Phi\rangle_{\mathrm{A}}^{\mathrm{new}} = \bigotimes_{i=1}^{\xi+1} \left(|\Phi\rangle_{\mathrm{A}^i}\right), \tag{S5}$$

and

$$|\Phi\rangle_{\mathrm{A}^i} = \overset{\mathrm{shift}}{\mathbf{U}} \left[\sum_{\mathbf{m}_{(i-1)N+1}^{iN}} \sum_{\mathbf{r'}_{(i-1)N+1}^{iN}} \sum_{\mathbf{a}_{(i-1)N+1}^{iN}} \left(\prod_{j=(i-1)N+1}^{iN} \sqrt{p_{r_j'} q_{a_j}}\right) \left(\bigotimes_{j=(i-1)N+1}^{iN} \left|m_j\right\rangle_{M_j} \left|r_j'\right\rangle_{A_j'} \left|a_j\right\rangle_{A_j''} \left|\psi^{\mathrm{imp}}_{\mathbf{r'}_{i-\xi}^i, \mathbf{a}_{i-\xi}^i}\right\rangle_{C_i}\right)\right], \tag{S6}$$

where the marker $\left|m_j\right\rangle_{M_j}$ satisfies

$$\left|m_j\right\rangle_{M_j} = \begin{cases} |1\rangle_{M_j} & j \in \{(i-1)N + i + n(\xi+1) | i \in \mathbb{N}, n \in \mathbb{N}_0, i + n(\xi+1) \leq N\} \\ |0\rangle_{M_j} & \text{otherwise} \end{cases}, \tag{S7}$$

which mark the *key generation rounds* mentioned in Proposition 1 with state 1, and the map $\overset{\mathrm{shift}}{\mathbf{U}}$ represents a process that uses the marker $\left|m_j\right\rangle_{M_j}$ to classify the local ancilla $\left|r_j'\right\rangle_{A_j'}$ either into the encoded qubit space A_j or to remain in the local ancilla space A_j', specifically

$$\overset{\mathrm{shift}}{\mathbf{U}} |1\rangle_{M_j} \left|r_j\right\rangle_{A_j'} = \left|r_j\right\rangle_{A_j}, \quad \overset{\mathrm{shift}}{\mathbf{U}} |0\rangle_{M_j} \left|r_j'\right\rangle_{A_j'} = \left|r_j'\right\rangle_{A_j'}. \tag{S8}$$

Thus, we can observe that there are N encoded qubits within the $\bigcup_{j=1}^{(\xi+1)N} A_j$ space, while there are ξN ancillas remaining in the $\bigcup_{j=1}^{(\xi+1)N} A'_j$ space. This observation can also be intuitively inferred from Fig. 1(B) in the main text. Or in another word, before the map $\overset{\text{shift}}{\mathbf{U}}$, the $\bigcup_{j=1}^{(\xi+1)N} A_j$ space is void and the $\bigcup_{j=1}^{(\xi+1)N} A'_j$ space has $(\xi+1)N$ non-void subspace A_j. And after the map $\overset{\text{shift}}{\mathbf{U}}$, the $\bigcup_{j=1}^{(\xi+1)N} A_j$ space has N non-void subspaces and the $\bigcup_{j=1}^{(\xi+1)N} A'_j$ space has N void subspace, and these subspaces transformed by the map is those in the *key generation rounds*, which satisfies $j \in \{(i-1)N + i + n(\xi+1) | i \in \mathbb{N}, n \in \mathbb{N}_0, i + n(\xi+1) \leq N\}$. Afterward, we perform a rearrangement, discarding the empty subspaces (as they hold no significance). This rearrangement results in $\bigcup_{j=1}^{(\xi+1)N} A_j \to \bigcup_{j=1}^{N} A_j$ and $\bigcup_{j=1}^{(\xi+1)N} A'_j \to \bigcup_{j=1}^{\xi N} A'_j$. Then we denote the mappings and inverse mappings of the indices under these two rearrangements as $f_{\text{rea}}(i)$, $f'_{\text{rea}}(i)$ and $f_{\text{rea}}^{-1}(i)$, $f'^{-1}_{\text{rea}}(i)$.

Even though the *new protocol* given by Eq. (S5) is an uncorrelated protocol, it still significantly differs from the common two-state SNS protocol. Furthermore, due to the lack of critical parameters, estimating its phase error rate remains challenging. To address this, we further constrain the security of the *original protocol* by introducing an *equivalent protocol* to analyze its security. By reorganizing Eq. (S5) to emphasize and reorder the encoded qubit space of the *key generation rounds*, we obtain that the entanglement-equivalent protocol of the new protocol satisfies

$$\begin{aligned} |\Phi\rangle_{\mathrm{A}}^{\text{new}} = \Bigg[& \sum_{\mathbf{r'}_1^{\xi N}} \sum_{\mathbf{a}_1^{(1+\xi)N}} \left(\prod_{i=1}^{\xi N} \sqrt{p_{r'_i}} \prod_{j=1}^{(1+\xi)N} \sqrt{q_{a_j}} \right) \left(\bigotimes_{i=1}^{\xi N} |r'_i\rangle_{A'_i} \bigotimes_{j=1}^{(1+\xi)N} |a_j\rangle_{A''_j} \right) \\ & \otimes \left[\sum_{\mathbf{r}_1^N} \left(\prod_{i=1}^{N} \sqrt{p_{r_i}} \right) \left(\bigotimes_{i=1}^{N} |r_i\rangle_{A_i} \left| \psi^{\text{imp}'}_{r_i, \mathbf{r'}(i,\xi), \mathbf{a}(i,\xi)} \right\rangle_{C'_i} \right) \right] \Bigg], \end{aligned} \tag{S9}$$

where $\mathbf{r'}(i,\xi) = \mathbf{r'}_{f'_{\text{rea}}(f_{\text{rea}}^{-1}(i)-\xi)}^{f'_{\text{rea}}(f_{\text{rea}}^{-1}(i)+\xi)}$ denotes the local ancilla in the previous ξ *physical rounds* and the following ξ *physical rounds* of the i-th *key generation round*, $\mathbf{a}(i,\xi) = \mathbf{a}_{f_{\text{rea}}^{-1}(i)-\xi}^{f_{\text{rea}}^{-1}(i)+\xi}$ denotes the system ancilla of the up mentioned *physical rounds* and the i-th *key generation round*, and $\left| \psi^{\text{imp}'}_{r_i, \mathbf{r'}(i,\xi), \mathbf{a}(i,\xi)} \right\rangle_{C'_i}$ denote the state send into the channel in the i-th *key generation round* and the following ξ *physical*

rounds, satisfies

$$\begin{aligned}\left|\psi^{\text{imp}'}_{r_i,\mathbf{r}'(i,\xi),\mathbf{a}(i,\xi)}\right\rangle_{C_i'} =&\left|\psi^{\text{imp}}_{r_{f_{\text{rea}}^{-1}(i)}\mathbf{r}'^{f'_{\text{rea}}(f_{\text{rea}}^{-1}(i)-1)}_{f'_{\text{rea}}(f_{\text{rea}}^{-1}(i)-\xi)},\mathbf{a}^{f_{\text{rea}}^{-1}(i)}_{f_{\text{rea}}^{-1}(i)-\xi}}\right\rangle_{C_{f_{\text{rea}}^{-1}(i)}} \\ &\bigotimes_{j=1}^{\xi}\left|\psi^{\text{imp}}_{\mathbf{r}'^{f'_{\text{rea}}(f_{\text{rea}}^{-1}(i)+j)}_{f'_{\text{rea}}(f_{\text{rea}}^{-1}(i)+1)}r_{f_{\text{rea}}^{-1}(i)}\mathbf{r}'^{f'_{\text{rea}}(f_{\text{rea}}^{-1}(i)-1)}_{f'_{\text{rea}}(f_{\text{rea}}^{-1}(i)-\xi+j)},\mathbf{a}^{f_{\text{rea}}^{-1}(i)+j}_{f_{\text{rea}}^{-1}(i)-\xi+j}}\right\rangle_{C_{f_{\text{rea}}^{-1}(i)+j}},\end{aligned} \tag{S10}$$

and where $\left|\psi^{\text{imp}}_{\mathbf{r}^i_{i-\xi},\mathbf{a}^i_{i-\xi}}\right\rangle_{C_i}$ is denoted in Eq. (S3).

As discussed in Proposition 2, the new protocol reveals all *physical rounds* except the *key generation rounds*. Furthermore, since the security of the *original protocol* is constrained by that of the *new protocol*, we can relax the assumptions on the *new protocol*. Therefore, we further disclose the ancilla in the space A_i'' for all *physical rounds* and assume that Alice sends additional quantum states into the channel. Thus, for any additional state $\left|\psi^{\text{add}}_{r_i,\mathbf{r}'(i,\xi),\mathbf{a}(i,\xi)}\right\rangle_{C_i''}$ sent into the channel, the security of protocol $|\Phi\rangle_{\text{A}}^{\text{new}}$ in Eq. (S9) can be guaranteed by protocol $|\Phi\rangle_{\text{A}}^{\text{new}_2}$, satisfying

$$\begin{aligned}|\Phi\rangle_{\text{A}}^{\text{new}_2} =&\left[\sum_{\mathbf{r}'^{\xi N}_1}\sum_{\mathbf{a}_1^{(1+\xi)N}}\left(\prod_{i=1}^{\xi N}\sqrt{p_{r_i'}}\prod_{j=1}^{(1+\xi)N}\sqrt{q_{a_j}}\right)\left(\bigotimes_{i=1}^{\xi N}\left|r_i'\right\rangle_{A_i'}\bigotimes_{j=1}^{(1+\xi)N}\left|a_j\right\rangle_{A_j''}\right)\right. \\ &\left.\otimes\left[\sum_{\mathbf{r}_1^N}\left(\prod_{i=1}^{N}\sqrt{p_{r_i}}\right)\left(\bigotimes_{i=1}^{N}|r_i\rangle_{A_i}\left|\psi^{\text{imp}'}_{r_i,\mathbf{r}'(i,\xi),\mathbf{a}(i,\xi)}\right\rangle_{C_i'}\left|\psi^{\text{add}}_{r_i,\mathbf{r}'(i,\xi),\mathbf{a}(i,\xi)}\right\rangle_{C_i''}\right)\right]\right].\end{aligned} \tag{S11}$$

In protocol $|\Phi\rangle_{\text{A}}^{\text{new}_2}$, we may isolate the terms related to the i-th coding ancilla choice r_i and denote it as

$$\begin{aligned}|\Phi\rangle_{\text{A,iso}\,r_i}^{\text{new}_2,i} =&\left[\sum_{\mathbf{r}'(i,\xi)}\sum_{\mathbf{a}(i,\xi)}\left(\prod_{j=f'_{\text{rea}}(f_{\text{rea}}^{-1}(i)-\xi)}^{j=f'_{\text{rea}}(f_{\text{rea}}^{-1}(i)+\xi)}\sqrt{p_{r_j'}}\prod_{k=f_{\text{rea}}^{-1}(i)-\xi}^{k=f_{\text{rea}}^{-1}(i)+\xi}\sqrt{p_{a_k}}\right)\left(\bigotimes_{j=f'_{\text{rea}}(f_{\text{rea}}^{-1}(i)-\xi)}^{j=f'_{\text{rea}}(f_{\text{rea}}^{-1}(i)+\xi)}\left|r_j'\right\rangle_{A_j'}\bigotimes_{k=f_{\text{rea}}^{-1}(i)-\xi}^{k=f_{\text{rea}}^{-1}(i)+\xi}|a_k\rangle_{A_k''}\right)\right. \\ &\left.\otimes\left|\psi^{\text{imp}'}_{r_i,\mathbf{r}'(i,\xi),\mathbf{a}(i,\xi)}\right\rangle_{C_i'}\left|\psi^{\text{add}}_{r_i,\mathbf{r}'(i,\xi),\mathbf{a}(i,\xi)}\right\rangle_{C_i''}\right].\end{aligned} \tag{S12}$$

According to Eq. (S10), we further define the vacuum state in the C_i' space as $|0\rangle_{C_i'}$, satisfying

$$|0\rangle_{C_i'} = |0\rangle_{C_{f_{\text{rea}}^{-1}(i)}}\bigotimes_{j=1}^{\xi}|0\rangle_{C_{f_{\text{rea}}^{-1}(i)+j}}. \tag{S13}$$

Since $\left|\psi^{\text{add}}_{r_i,\mathbf{r}'(i,\xi),\mathbf{a}(i,\xi)}\right\rangle_{C_i''}$ is arbitrary, we can transfer some of its phase to $\left|\psi^{\text{imp}'}_{r_i,\mathbf{r}'(i,\xi),\mathbf{a}(i,\xi)}\right\rangle_{C_i'}$, to form a new state $\left|\psi^{\text{imp}''}_{r_i,\mathbf{r}'(i,\xi),\mathbf{a}(i,\xi)}\right\rangle_{C_i'}$ such that $\left|\psi^{\text{imp}'}_{r_i,\mathbf{r}'(i,\xi),\mathbf{a}(i,\xi)}\right\rangle_{C_i'}\left|\psi^{\text{add}}_{r_i,\mathbf{r}'(i,\xi),\mathbf{a}(i,\xi)}\right\rangle_{C_i''} = \left|\psi^{\text{imp}''}_{r_i,\mathbf{r}'(i,\xi),\mathbf{a}(i,\xi)}\right\rangle_{C_i'}\left|\psi^{\text{add}'}_{r_i,\mathbf{r}'(i,\xi),\mathbf{a}(i,\xi)}\right\rangle_{C_i''}$

, $\left|\left\langle \psi^{\mathrm{imp}'}_{r_i,\mathbf{r}'(i,\xi),\mathbf{a}(i,\xi)} \middle| \psi^{\mathrm{imp}''}_{r_i,\mathbf{r}'(i,\xi),\mathbf{a}(i,\xi)} \right\rangle_{C'_i}\right| = \left|\left\langle \psi^{\mathrm{add}}_{r_i,\mathbf{r}'(i,\xi),\mathbf{a}(i,\xi)} \middle| \psi^{\mathrm{add}'}_{r_i,\mathbf{r}'(i,\xi),\mathbf{a}(i,\xi)} \right\rangle_{C''_i}\right| = 1$, and $\left\langle 0 \middle| \psi^{\mathrm{imp}''}_{r_i,\mathbf{r}'(i,\xi),\mathbf{a}(i,\xi)} \right\rangle_{C'_i}$ is real and positive. It is also worth noting that after this step, $\left|\psi^{\mathrm{add}'}_{r_i,\mathbf{r}'(i,\xi),\mathbf{a}(i,\xi)}\right\rangle_{C''_i}$ still retains its arbitrariness, both in terms of phase and magnitude. Subsequently, for the sake of computational simplicity, we further define two intermediate states, $|\Phi\rangle^{\mathrm{new},i}_{\mathrm{A,iso}\,r_i}$ and $|\Phi\rangle^{\mathrm{new},i}_{\mathrm{A,vac}}$, satisfy

$$\begin{aligned}
|\Phi\rangle^{\mathrm{new},i}_{\mathrm{A,iso}\,r_i} =& \Bigg[\sum_{\mathbf{r}'(i,\xi)}\sum_{\mathbf{a}(i,\xi)}\left(\prod_{j=f'_{\mathrm{rea}}(f^{-1}_{\mathrm{rea}}(i)-\xi)}^{j=f'_{\mathrm{rea}}(f^{-1}_{\mathrm{rea}}(i)+\xi)}\sqrt{p_{r'_j}}\prod_{k=f^{-1}_{\mathrm{rea}}(i)-\xi}^{k=f^{-1}_{\mathrm{rea}}(i)+\xi}\sqrt{p_{a_k}}\right)\left(\bigotimes_{j=f'_{\mathrm{rea}}(f^{-1}_{\mathrm{rea}}(i)-\xi)}^{j=f'_{\mathrm{rea}}(f^{-1}_{\mathrm{rea}}(i)+\xi)}\left|r'_j\right\rangle_{A'_j}\bigotimes_{k=f^{-1}_{\mathrm{rea}}(i)-\xi}^{k=f^{-1}_{\mathrm{rea}}(i)+\xi}|a_k\rangle_{A''_k}\right)\\
&\otimes\left|\psi^{\mathrm{imp}''}_{r_i,\mathbf{r}'(i,\xi),\mathbf{a}(i,\xi)}\right\rangle_{C'_i}\Bigg],\\
|\Phi\rangle^{\mathrm{new},i}_{\mathrm{A,vac}} =& \Bigg[\sum_{\mathbf{r}'(i,\xi)}\sum_{\mathbf{a}(i,\xi)}\left(\prod_{j=f'_{\mathrm{rea}}(f^{-1}_{\mathrm{rea}}(i)-\xi)}^{j=f'_{\mathrm{rea}}(f^{-1}_{\mathrm{rea}}(i)+\xi)}\sqrt{p_{r'_j}}\prod_{k=f^{-1}_{\mathrm{rea}}(i)-\xi}^{k=f^{-1}_{\mathrm{rea}}(i)+\xi}\sqrt{p_{a_k}}\right)\left(\bigotimes_{j=f'_{\mathrm{rea}}(f^{-1}_{\mathrm{rea}}(i)-\xi)}^{j=f'_{\mathrm{rea}}(f^{-1}_{\mathrm{rea}}(i)+\xi)}\left|r'_j\right\rangle_{A'_j}\bigotimes_{k=f^{-1}_{\mathrm{rea}}(i)-\xi}^{k=f^{-1}_{\mathrm{rea}}(i)+\xi}|a_k\rangle_{A''_k}\right)|0\rangle_{C'_i}\Bigg].
\end{aligned}\tag{S14}$$

Thus, due to Assumption 4, which further leads to Eq. (S4), the two intermediate states defined in Eq. (S14) satisfies

$$\begin{aligned}
\langle\Phi|^{\mathrm{new},i}_{\mathrm{A,iso}\,r_i}|\Phi\rangle^{\mathrm{new},i}_{\mathrm{A,vac}} =& \left|\sum_{\mathbf{r}'(i,\xi)}\sum_{\mathbf{a}(i,\xi)}\left(\prod_{j=f'_{\mathrm{rea}}(f^{-1}_{\mathrm{rea}}(i)-\xi)}^{j=f'_{\mathrm{rea}}(f^{-1}_{\mathrm{rea}}(i)+\xi)}p_{r'_j}\prod_{k=f^{-1}_{\mathrm{rea}}(i)-\xi}^{k=f^{-1}_{\mathrm{rea}}(i)+\xi}p_{a_k}\right)\left\langle\psi^{\mathrm{imp}''}_{r_i,\mathbf{r}'(i,\xi),\mathbf{a}(i,\xi)}\middle|0\right\rangle_{C'_i}\right|\\
\geq& \sum_{\mathbf{r}'(i,\xi)}\sum_{\mathbf{a}(i,\xi)}\left(\prod_{j=f'_{\mathrm{rea}}(f^{-1}_{\mathrm{rea}}(i)-\xi)}^{j=f'_{\mathrm{rea}}(f^{-1}_{\mathrm{rea}}(i)+\xi)}p_{r'_j}\prod_{k=f^{-1}_{\mathrm{rea}}(i)-\xi}^{k=f^{-1}_{\mathrm{rea}}(i)+\xi}p_{a_k}\right)\left(\sqrt{V^{\mathrm{A}}_{r_i}}\prod_{j=f'_{\mathrm{rea}}(f^{-1}_{\mathrm{rea}}(i)-\xi)}^{j=f'_{\mathrm{rea}}(f^{-1}_{\mathrm{rea}}(i)+\xi)}\sqrt{V^{\mathrm{A}}_{r'_i}}\right)\\
=&\sqrt{V^{\mathrm{A}}_{r_i}}\left(p_0\sqrt{V^{\mathrm{A}}_0}+p_1\sqrt{V^{\mathrm{A}}_1}\right)^{\xi} =: \sqrt{V^{\mathrm{A},\xi}_{r_i}}.
\end{aligned}\tag{S15}$$

Because Eq. (S15) satisfies for both $r_i = 0$ and 1, we can find that

$$\left|\langle\Phi|^{\mathrm{new},i}_{\mathrm{A,iso}\,0}|\Phi\rangle^{\mathrm{new},i}_{\mathrm{A,iso}\,1}\right| \geq \sqrt{V^{\mathrm{A},\xi}_0 V^{\mathrm{A},\xi}_1} - \sqrt{\left(1-V^{\mathrm{A},\xi}_0\right)\left(1-V^{\mathrm{A},\xi}_1\right)}.\tag{S16}$$

Further, from Eq. (S12) and Eq. (S14), we can calculate that

$$\begin{aligned}
&\langle\Phi|^{\mathrm{new}_2,i}_{\mathrm{A,iso}\,0}|\Phi\rangle^{\mathrm{new}_2,i}_{\mathrm{A,iso}\,1}\\
=&\sum_{\mathbf{r}'(i,\xi)}\sum_{\mathbf{a}(i,\xi)}\left(\prod_{j=f'_{\mathrm{rea}}(f^{-1}_{\mathrm{rea}}(i)-\xi)}^{j=f'_{\mathrm{rea}}(f^{-1}_{\mathrm{rea}}(i)+\xi)}p_{r'_j}\prod_{k=f^{-1}_{\mathrm{rea}}(i)-\xi}^{k=f^{-1}_{\mathrm{rea}}(i)+\xi}p_{a_k}\right)\left\langle\psi^{\mathrm{imp}''}_{0,\mathbf{r}'(i,\xi),\mathbf{a}(i,\xi)}\middle|\psi^{\mathrm{imp}''}_{1,\mathbf{r}'(i,\xi),\mathbf{a}(i,\xi)}\right\rangle_{C'_i}\left\langle\psi^{\mathrm{add}'}_{0,\mathbf{r}'(i,\xi),\mathbf{a}(i,\xi)}\middle|\psi^{\mathrm{add}'}_{1,\mathbf{r}'(i,\xi),\mathbf{a}(i,\xi)}\right\rangle_{C''_i},
\end{aligned}\tag{S17}$$

and

$$\langle\Phi|^{\mathrm{new},i}_{\mathrm{A,iso}\,0}|\Phi\rangle^{\mathrm{new},i}_{\mathrm{A,iso}\,1} = \sum_{\mathbf{r}'(i,\xi)}\sum_{\mathbf{a}(i,\xi)}\left(\prod_{j=f'_{\mathrm{rea}}(f^{-1}_{\mathrm{rea}}(i)-\xi)}^{j=f'_{\mathrm{rea}}(f^{-1}_{\mathrm{rea}}(i)+\xi)}p_{r'_j}\prod_{k=f^{-1}_{\mathrm{rea}}(i)-\xi}^{k=f^{-1}_{\mathrm{rea}}(i)+\xi}p_{a_k}\right)\left\langle\psi^{\mathrm{imp}''}_{0,\mathbf{r}'(i,\xi),\mathbf{a}(i,\xi)}\middle|\psi^{\mathrm{imp}''}_{1,\mathbf{r}'(i,\xi),\mathbf{a}(i,\xi)}\right\rangle_{C'_i}.\tag{S18}$$

Since $\left|\left\langle\psi^{\mathrm{add}'}_{0,\mathbf{r}'(i,\xi),\mathbf{a}(i,\xi)}\middle|\psi^{\mathrm{add}'}_{1,\mathbf{r}'(i,\xi),\mathbf{a}(i,\xi)}\right\rangle_{C''_i}\right| \le 1$, combine with Eq. (S16) we can select a specific set of $\left|\psi^{\mathrm{add}'}_{r_i,\mathbf{r}'(i,\xi),\mathbf{a}(i,\xi)}\right\rangle_{C''_i}$ such that

$$\langle\Phi|^{\mathrm{new}_2,i}_{\mathrm{A,iso}\,0}\,|\Phi\rangle^{\mathrm{new}_2,i}_{\mathrm{A,iso}\,1} = \sqrt{V_0^{\mathrm{A},\xi}V_1^{\mathrm{A},\xi}} - \sqrt{\left(1-V_0^{\mathrm{A},\xi}\right)\left(1-V_1^{\mathrm{A},\xi}\right)}. \tag{S19}$$

To reiterate, in protocol $|\Phi\rangle^{\mathrm{new}_2}_{\mathrm{A}}$, all quantum states in spaces other than the encoded qubit space $\bigcup_{j=1}^{N} A_j$, including ancillary particles, are sent into the channel. Therefore, performing a unitary mapping in the remaining spaces does not affect the security of the protocol. Specifically, we construct an *equivalent protocol* $|\Phi\rangle^{\mathrm{equ}}_{\mathrm{A}}$, satisfies

$$\begin{aligned}
|\Phi\rangle^{\mathrm{equ}}_{\mathrm{A}} &= \left(\prod_{j=1}^{N}\mathbf{U}_j\right)|\Phi\rangle^{\mathrm{new}_2}_{\mathrm{A}}\\
&= \left[\sum_{\mathbf{r}'^{\xi N}_1}\sum_{\mathbf{a}^{(1+\xi)N}_1}\left(\prod_{i=1}^{\xi N}\sqrt{p_{r'_i}}\prod_{j=1}^{(1+\xi)N}\sqrt{q_{a_j}}\right)\left(\bigotimes_{i=1}^{\xi N}|r'_i\rangle_{A'_i}\bigotimes_{i=1}^{(1+\xi)N}|a_i\rangle_{A''_i}\right)\right.\\
&\quad\left.\otimes\left[\sum_{\mathbf{r}^N_1}\left(\prod_{i=1}^{N}\sqrt{p_{r_i}}\right)\left(\bigotimes_{i=1}^{N}|r_i\rangle_{A_i}\,|\psi^{\mathrm{equ}}_{r_i}\rangle_{C'''_i}\right)\right]\right],
\end{aligned} \tag{S20}$$

where $\mathbf{U}_i$ si an unitary mapping from the space $\bigcup_{j=f'_{\mathrm{rea}}(f^{-1}_{\mathrm{rea}}(i)-\xi)}^{j=f'_{\mathrm{rea}}(f^{-1}_{\mathrm{rea}}(i)+\xi)} A'_j \bigcup_{k=f^{-1}_{\mathrm{rea}}(i)-\xi}^{k=f^{-1}_{\mathrm{rea}}(i)+\xi} A''_k \cup C'_i \cup C''_i$ into $\bigcup_{j=f'_{\mathrm{rea}}(f^{-1}_{\mathrm{rea}}(i)-\xi)}^{j=f'_{\mathrm{rea}}(f^{-1}_{\mathrm{rea}}(i)+\xi)} A'_j \bigcup_{k=f^{-1}_{\mathrm{rea}}(i)-\xi}^{k=f^{-1}_{\mathrm{rea}}(i)+\xi} A''_k \cup C'''_i$, satisfies

$$\begin{aligned}
&\mathbf{U}_i\,|\Phi\rangle^{\mathrm{new}_2,i}_{\mathrm{A,iso}\,r_i}\\
&= \left[\sum_{\mathbf{r}'(i,\xi)}\sum_{\mathbf{a}(i,\xi)}\left(\prod_{j=f'_{\mathrm{rea}}(f^{-1}_{\mathrm{rea}}(i)-\xi)}^{j=f'_{\mathrm{rea}}(f^{-1}_{\mathrm{rea}}(i)+\xi)}\sqrt{p_{r'_j}}\prod_{k=f^{-1}_{\mathrm{rea}}(i)-\xi}^{k=f^{-1}_{\mathrm{rea}}(i)+\xi}\sqrt{p_{a_k}}\right)\left(\bigotimes_{j=f'_{\mathrm{rea}}(f^{-1}_{\mathrm{rea}}(i)-\xi)}^{j=f'_{\mathrm{rea}}(f^{-1}_{\mathrm{rea}}(i)+\xi)}\left|r'_j\right\rangle_{A'_j}\bigotimes_{k=f^{-1}_{\mathrm{rea}}(i)-\xi}^{k=f^{-1}_{\mathrm{rea}}(i)+\xi}|a_k\rangle_{A''_k}\right)\otimes\left|\psi^{\mathrm{equ}}_{r_i}\right\rangle_{C'''_i}\right].
\end{aligned} \tag{S21}$$

From Eq. (S21), we can see that $\mathbf{U}_i$ exists if and only if

$$\langle\Phi|^{\mathrm{new}_2,i}_{\mathrm{A,iso}\,0}\,|\Phi\rangle^{\mathrm{new}_2,i}_{\mathrm{A,iso}\,1} = \sum_{\mathbf{r}'(i,\xi)}\sum_{\mathbf{a}(i,\xi)}\left(\prod_{j=f'_{\mathrm{rea}}(f^{-1}_{\mathrm{rea}}(i)-\xi)}^{j=f'_{\mathrm{rea}}(f^{-1}_{\mathrm{rea}}(i)+\xi)}p_{r'_j}\prod_{k=f^{-1}_{\mathrm{rea}}(i)-\xi}^{k=f^{-1}_{\mathrm{rea}}(i)+\xi}p_{a_k}\right)\left\langle\psi^{\mathrm{equ}}_0\middle|\psi^{\mathrm{equ}}_1\right\rangle_{C'''_i} = \left\langle\psi^{\mathrm{equ}}_0\middle|\psi^{\mathrm{equ}}_1\right\rangle_{C'''_i}. \tag{S22}$$

Without loss of generality, we assume that

$$\left|\psi^{\mathrm{equ}}_0\right\rangle_{C'''_i} = |0\rangle\,,\ \left|\psi^{\mathrm{equ}}_1\right\rangle_{C'''_i} = \left|\mu_{\mathrm{equ}}\right\rangle, \tag{S23}$$

where $|0\rangle$ is the vacuum state and $\left|\mu_{\text{equ}}\right\rangle$ is the coherent state with an average number of photons equals to μ_{equ}. Combine Eqs. (S19), (S22) and (S23), we can calculate that μ_{equ} satisfies

$$\mathrm{e}^{-\mu_{\text{equ}}} = \left[\sqrt{V_0^{\mathrm{A},\xi} V_1^{\mathrm{A},\xi}} - \sqrt{\left(1 - V_0^{\mathrm{A},\xi}\right)\left(1 - V_1^{\mathrm{A},\xi}\right)}\right]^2 . \tag{S24}$$

Furthermore, observing that in protocol $|\Phi\rangle_{\mathrm{A}}^{\text{equ}}$ as defined in Eq. (S20), r' and a no longer play any role, we simplify the *equivalent protocol* $|\Phi\rangle_{\mathrm{A}}^{\text{equ}}$ by removing them. The final *equivalent protocol* then satisfies

$$|\Phi\rangle_{\mathrm{A}}^{\text{equ}} = \left[\sum_{\mathbf{r}_1^N}\left(\prod_{i=1}^{N}\sqrt{p_{r_i}}\right)\left(\bigotimes_{i=1}^{N}|r_i\rangle_{A_i}\left|\psi_{r_i}^{\text{equ}}\right\rangle_{C_i'''}\right)\right] . \tag{S25}$$

The above analysis provides a proof that, for general values of ξ, protocol $|\Phi\rangle_{\mathrm{A}}^{\text{equ}}$ satisfies Eq. (S25) ensures the security of $|\Phi\rangle_{\mathrm{A}}$.
